# Live cell tracking of macrophage efferocytosis during *Drosophila* embryo development in vivo

**DOI:** 10.1126/science.abl4430

**Published:** 2022-03-10

**Authors:** Michael H. Raymond, Andrew J. Davidson, Yi Shen, Daniel R. Tudor, Christopher D. Lucas, Sho Morioka, Justin S.A. Perry, Julia Krapivkina, David Perrais, Linus J. Schumacher, Robert E. Campbell, Will Wood, Kodi S. Ravichandran

**Affiliations:** 1 Center for Cell Clearance, University of Virginia, Charlottesville, VA, USA; 2 Department of Microbiology, Immunology, and Cancer Biology, University of Virginia, Charlottesville, VA, USA; 3 Neuroscience Graduate Program, University of Virginia, Charlottesvill, VA, USA; 4 Department of Medicine, Division of Nephrology and CIIR, University of Virginia, Charlottesville, VA, USA; 5 Centre for Inflammation Research, University of Edinburgh, Edinburgh BioQuarter, Edinburgh, UK; 6 Centre for Regenerative Medicine, University of Edinburgh, Edinburgh BioQuarter, Edinburgh, UK; 7 Immunology Program, Memorial Sloan Kettering Cancer Center, New York, NY 10065; 8 Department of Chemistry, University of Alberta, Edmonton, Canada; 9 University of Bordeaux, CNRS, Interdisciplinary Institute for Neuroscience, IINS, UMR 5297, Bordeaux, France; 10 VIB/UGent Inflammation Research Centre, and Biomedical Molecular Biology, Ghent University, Belgium; 11 Division of Immunobiology, Department of Pathology and Immunology, Washington University School of Medicine, St. Louis, MO, USA

## Abstract

Apoptosis of cells and their subsequent removal via efferocytosis occurs in nearly all tissues during development, homeostasis, and disease. However, it has been difficult to track cell death and subsequent corpse removal in vivo. Here, we developed a genetically encoded fluorescent reporter, CharON, that could track emerging apoptotic cells and their efferocytic clearance by phagocytes. Using *Drosophila* expressing CharON, we uncovered multiple qualitative and quantitative features of coordinated clearance of apoptotic corpses during embryonic development. To confront high rate of emerging apoptotic corpses, the macrophages displayed heterogeneity in engulfment, with some efferocytic macrophages carrying high corpse burden. However, overburdened macrophages were compromised in clearing wound debris, revealing an inherent vulnerability. These findings reveal known and unexpected features of apoptosis and macrophage efferocytosis in vivo.

Efferocytosis (the uptake and degradation of apoptotic cells by phagocytes) is essential for tissue development, homeostasis and resolution of inflammation (*1,2*). Previous work has characterized efferocytic receptors on phagocytes and their corresponding ligands on dying cells; however tracking efferocytosis *in vivo* has proven a longstanding challenge (*3,4,5*). Even detecting apoptotic cells in vivo is difficult due to their swift removal and the lack of tools to track them as they emerge.

To track both apoptosis and efferocytosis in vivo, we engineered two probes. For the apoptosis reporter, we tested multiple designs where the cleavage by executioner caspases 3 and 7 induced fluorescence of a green fluorescent protein (GFP) (Figures 1A, S1A-G, S2A-D, see Methods) (*6,7,8,9*). A limitation of GFP-based probes is photo-quenching in the acidic environment of the endosomes/lysosomes. To circumvent this, we engineered pH-CaspGFP to be pH-tolerant through mutation (Q204H), based on the parent GC3ai molecule (Figure 1B) (*10*). pH-CaspGFP faithfully reported apoptosis after different triggers and in different cell types (Figures S1A-G, S2A-D).

To detect corpse acidification and digestion within phagosomes, we engineered a new red-fluorescent pH sensor, pHlorina, which exhibits increasing fluorescence with decreasing pH (Figures 1C, S3A-E, see Methods). To assess pHlorina during efferocytosis, we stably transduced Jurkat cells, stimulated apoptosis, and incubated with mouse J774 macrophages (Figures 1D and S4A). Upon corpse internalization, a 3.4 fold increase in pHlorina fluorescence was detected (Figure 1D), and this was inhibited by the acidification inhibitor Bafilomycin or *Rubicon* knockdown (Figures 1D, and S4B-S4C). Thus, pHlorina can track internalized apoptotic corpses during acidification/digestion within phagocytes.

Next, we combined the above two probes to generate ‘CharON’ (‘ **C** aspase and p **H** **A** ctivated **R** eporter, Fluorescence **ON** ) referring to *Charon*, who in Greek mythology ferries the deceased across the river of the dead (Figure 2A, see Methods). Through CharON, an apoptotic cell first turns GFP+. Following engulfment by a phagocyte and acidification within the phagolysosome, the intensity of pHlorina increases as the GFP gradually quenches (Figure 2A). When apoptotic CharON expressing Jurkat cells were mixed with BFP^+^ mouse J774 macrophages or mouse bone marrow derived macrophages, CharON permitted all the stages of efferocytosis to be visualised (Figures S4D-S4E, Video S1). Both fluorescent components of CharON exhibited wide dynamic ranges (Figure 2B), with a 17-fold increase in average pHlorina/pH-CaspGFP ratio within 2 hours of corpse internalization (Figure 2B). Furthermore, CharON visualised successive efferocytic events, wherein macrophages engulfed multiple corpses (Figure S4F, Video S2). Thus, CharON is a powerful new tool for tracking apoptosis and efferocytosis.

To track apoptosis and efferocytosis in vivo, we generated CharON transgenic *Drosophila*. During mid-late *Drosophila* embryogenesis (stages 12-16), there is a wave of apoptosis in the developing Central Nervous System (CNS) (*13, 14*). Through CharON, developmental apoptosis and efferocytosis were visualised throughout the CNS (Figures 2C, S5A-D and Video S3). The apoptotic burden in embryonic *Drosophila* CNS is shared by the phagocytic glia and the dispersing ventral hemocytes (macrophages) (Figures 2C and S6A) (*15, 17*). The highly motile macrophages, disperse through the hemocoel (‘blood-cavity’), clearing apoptosis at the CNS interface (*16, 18*). Efferocytosis deep within the CNS was sporadic, representing constant, autonomous engulfment by phagocytic, non-motile glia, and conferred a weak trend for increasing CharON acidic fluorescence (Figure 2D). In contrast, the macrophage-mediated efferocytosis at the CNS interface was visualized as a choreographed wave of apoptosis and efferocytosis, yielding a strong increase in pHlorina signal (Figure 2E, Video S3). CharON also highlighted differences in phagosome size between phagocytic glia and macrophages (Figures S5E-F), with more ‘compacted’ corpse sizes in macrophages.

We focused on macrophage efferocytosis due to its synchronised pattern of CharON signal and the importance of macrophages in clearing cellular debris during human development and disease. A macrophage marker was combined with CharON to visualize macrophage-mediated efferocytosis in vivo (Video S4). Although the GFP signal of the macrophage marker overlapped with the pHCaspGFP of CharON, apoptotic cells were easily distinguished due to differences in morphologies and signal intensity (Video S4). We also used an mCherry macrophage marker to visualize early interactions between phagocyte and apoptotic corpses (Figure S6B). CharON permitted the full efferocytic program to be observed in vivo, including apoptosis, macrophage recruitment and target binding (“Find Me”), internalization (“Eat Me”) and corpse acidification/degradation (“Digest Me”) (Figures 3A, S6C and Video S5). Quantification of pHlorina-positive corpses within macrophages revealed that corpse burden peaked after dispersal (stage 15, post-clearance) (Figures 3B-3C). It was only after an initial lag that internalized corpses increased pHlorina intensity and decreased in size (i.e. degradation), implying macrophage uptake of corpses is unrestrained and not always synchronized with acidification/degradation (Figures 3D-3E).

Tracking macrophages during efferocytosis revealed that macrophages sensed and migrated up to 8% of the embryo length towards apoptosis, regardless of pre-existing corpse burden (Figures S6D-F) (*19*). Furthermore, multiple macrophages were often required to clear a single, fragmenting apoptotic corpse (Video S6). Interestingly, the recruitment and uptake of apoptotic corpses took less time (~5-7 min) than the time required for maximal corpse acidification (~25 min), suggesting corpse degradation could be rate limiting if this step is necessary for further uptake of additional apoptotic corpses (Figures 3A and S6C). Interestingly, macrophages maintained their high motility throughout efferocytosis and readily moved toward and engulfed successive corpses before acidification of their existing or newly acquired corpse (Figures S6D-F, Video S4). The first, ‘pioneer’ macrophages (stages 11/12), which initially disperse from the head region, were confronted by a dense field of apoptotic corpses (Figure S7A, Video S4). The rapid uptake of these corpses by the dispersing macrophages resulted in dramatic disparities in macrophage corpse burden (Figures 4A and S7B-C). We classified this heterogeneity between macrophages as having either no, low (1-3 corpses), medium (4-6 corpses) or high (≥7 corpses) burden (Figure 4B). Remarkable examples of macrophages with extreme burden (≥10 corpses) were also evident. Interestingly, high burden eventually led to increased corpse acidification, suggesting efferocytic adaptation, as shown previously (Figure S7D) (*20, 21, 22*).

These data suggested that when a high concentration of apoptotic cells emerge, unrestrained uptake ensures rapid clearance, but results in unequal corpse burden among macrophages. We tested this concept using agent-based modelling of macrophage efferocytosis, the parameters for which were derived from our in vivo observations (Figures S8A-B, see Methods). When virtual phagocytes and corpses were randomly distributed, corpse capacity limits exponentially increased clearance time (Figures 4C and S8C, Video S7). Conversely, releasing the phagocytes from strict consumption limits led to rapid clearance *in silico*, at the expense of unequal corpse burden among the macrophages. A similar pattern emerged from simulations where phagocytes chemotaxed through a field of corpses, wherein a strict consumption limit again impaired complete clearance (Figures S8D-E, Video S7).

We next explored consequences of varying corpse burden for in vivo macrophage behavior. Tracking of macrophages post-clearance (stage 15) demonstrated that corpse burden did not affect their basal motility, despite low and high corpse burdens representing different ‘physical’ loads (Figure S9A). Similarly, macrophages with high corpse burden readily migrated toward laser-induced wounds; further, there was no significant difference between responding and non-responding macrophages in terms of prior apoptotic corpse burden (Figures S9A-D, S10A, Video S8).

Importantly, laser-induced wounds in *Drosophila* embryos are entirely necrotic, as demonstrated by the lack of pH-CaspGFP fluorescence (Video S9). Macrophage-mediated clearance of necrotic debris was observed as fluorescence-negative particle uptake (i.e. lacking pH-CaspGFP signal), against the GFP-labelled macrophages. Although recruited comparably, macrophages with high apoptotic corpse burden exhibit an impaired ability to engulf necrotic debris at the wound, implying a ‘phagocytic satiety’ (Figure 4D). While high/extreme corpse burden compromises subsequent inflammatory efferocytosis, the heterogeneity between macrophages in corpse burden leads to sufficient macrophages with low corpse burden to ensure clearance.

In many pathologies, macrophages function in a background of increased cell death and therefore are bound to carry a higher corpse burden. To model this, we used the *repo* mutant (which lacks phagocytic glia and their efferocytic contribution), which increased the corpse burden on the macrophages (Figures 4E-4F and S10B) (*23, 24, 25*). This resulted in more macrophages with high and extreme corpse burdens and a near absence of macrophages with low burden. Next, using DRAQ7 dye to label the laser-induced necrotic corpses, we visualised inflammation in wild-type and *repo* embryos through 3-colour imaging. While *repo* mutant macrophages have impaired inflammatory chemotaxis, sufficient macrophages are recruited to analyze efferocytosis (*25*). Wild-type macrophages cleared all DRAQ7 labelled necrotic corpses within 30-60 mins (Figure 4G, Video S9). In contrast, overburdened *repo* macrophages struggled to engulf necrotic debris, even when directly contacting the wound (Figures 4G and S4C, Video S9).

Finally, CharON allowed us to track the fate of apoptotic and necrotic cargo within the same macrophage. Remarkably, rapid acidification of necrotic corpses sometimes occurred through fusion with phagolysosomes containing an apoptotic corpse (Figure 4H, Video S10). This suggested that macrophages may rapidly degrade necrotic debris to alleviate high corpse burden during times of heightened efferocytosis, such as inflammation.

As with advancements in genetically-encoded tools for the study of cellular function, we envision CharON proving instrumental in dissecting efferocytosis in vivo (*26, 27*). Whether macrophages arrest after they encounter, or ingest, an apoptotic cell has been debated (*20, 21, 22, 29*). Using CharON, we tracked developmental apoptosis and efferocytosis within the *Drosophila* embryo, in real-time. However, even macrophages which had recently engulfed multiple corpses remained highly motile and phagocytic, implying engulfment and motility are not mutually exclusive. Furthermore, we propose that macrophages prioritise unrestrained corpse uptake, as opposed to corpse degradation, to ensure the rapid clearance of extensive apoptosis (e.g. during development). While this results in variable (including extreme) corpse burdens, this efferocytic strategy undoubtedly reduces the risk of uncleared apoptotic corpses undergoing secondary necrosis. Furthermore, enforced equal sharing of corpses would undoubtedly require additional regulatory complexity.

In many human inflammatory diseases, there is increased and diverse forms of cell death, which can perturb efferocytosis (*1, 30*). It is unknown whether the same macrophage can engulf both apoptotic and necrotic corpses in vivo. In our model, we observed macrophages filled with apoptotic corpses seamlessly transition to clearing necrotic debris. However, we found overburdened macrophages were severely impaired in their ability to engulf further necrotic debris. Thus, the efferocytic strategy adopted by macrophages, which maximises clearance under homeostasis, may be counter-productive during pathology and ultimately exacerbate disease.

## Supplementary Material

Supplementary Data

## Figures and Tables

**Fig. 1:**
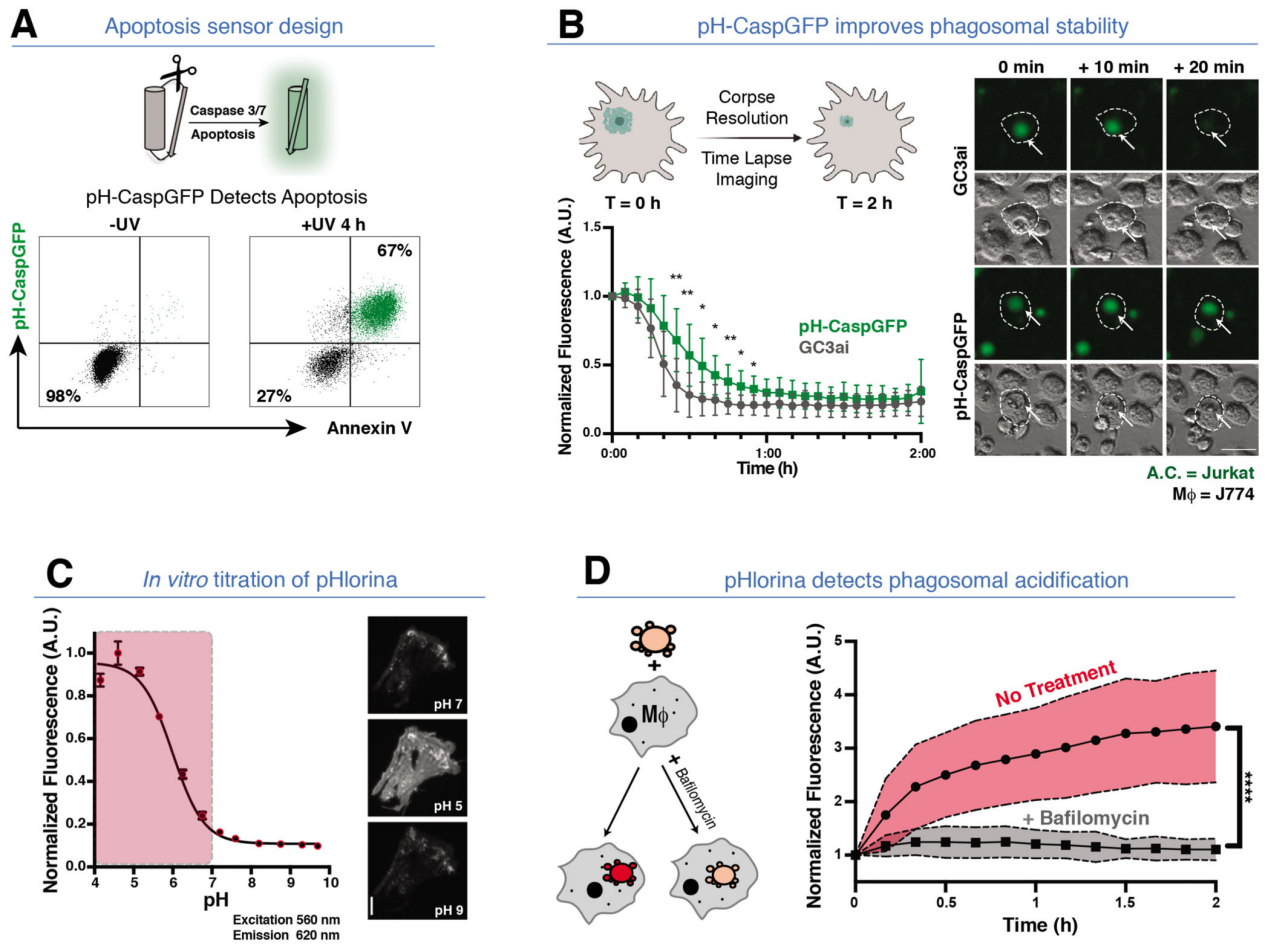
Engineering a pH-Stable apoptosis reporter and an RFP pH sensor. **(A)** Apoptosis sensor design. (Top) Upon apoptosis, a Caspase-3/7 linker is cleaved, promoting GFP fluorescence. (Bottom) GFP/Annexin V positivity of pH-CaspGFP expressing Jurkat cells ± apoptosis (4 hours post UV-C). **(B)** pH-CaspGFP exhibits improved phagosomal stability. (Top left) Schematic of in vitro engulfment assay: GC3ai or pH-GC3ai apoptotic Jurkat cells were co-cultured with J774 macrophages. (Bottom left) Upon internalization, GFP fluorescence intensity was tracked. (Right) Time-Lapse images of (top) GC3ai or (bottom) pH-CaspGFP apoptotic Jurkat cells engulfed by J774 Macrophages (arrows). Three independent experiments, Two-Way ANOVA, ** =p<.0021, * =p<.0332. **(C)** In vitro validation of pHlorina. (Left) pH titration of purified pHlorina from pH 4-10. (excitation/emission =560 nm/620 nm). (Right) pHlorina fluorescence at pH 7.0, pH 5.0 and pH 9.0. Three independent experiments. **(D)** pHlorina detects phagosomal acidification during efferocytosis. (Left) Schematic of in vitro engulfment assay to track pHlorina fluorescence during efferocytosis ± bafilomycin (to inhibit phagosomal acidification). (Right) pHlorina signal in apoptotic Jurkat cells post-engulfment by J774 macrophages ± bafilomycin. Three independent experiments, Unpaired t-Test, **** = p<.0001. All scale bars =50 μm.

**Fig. 2:**
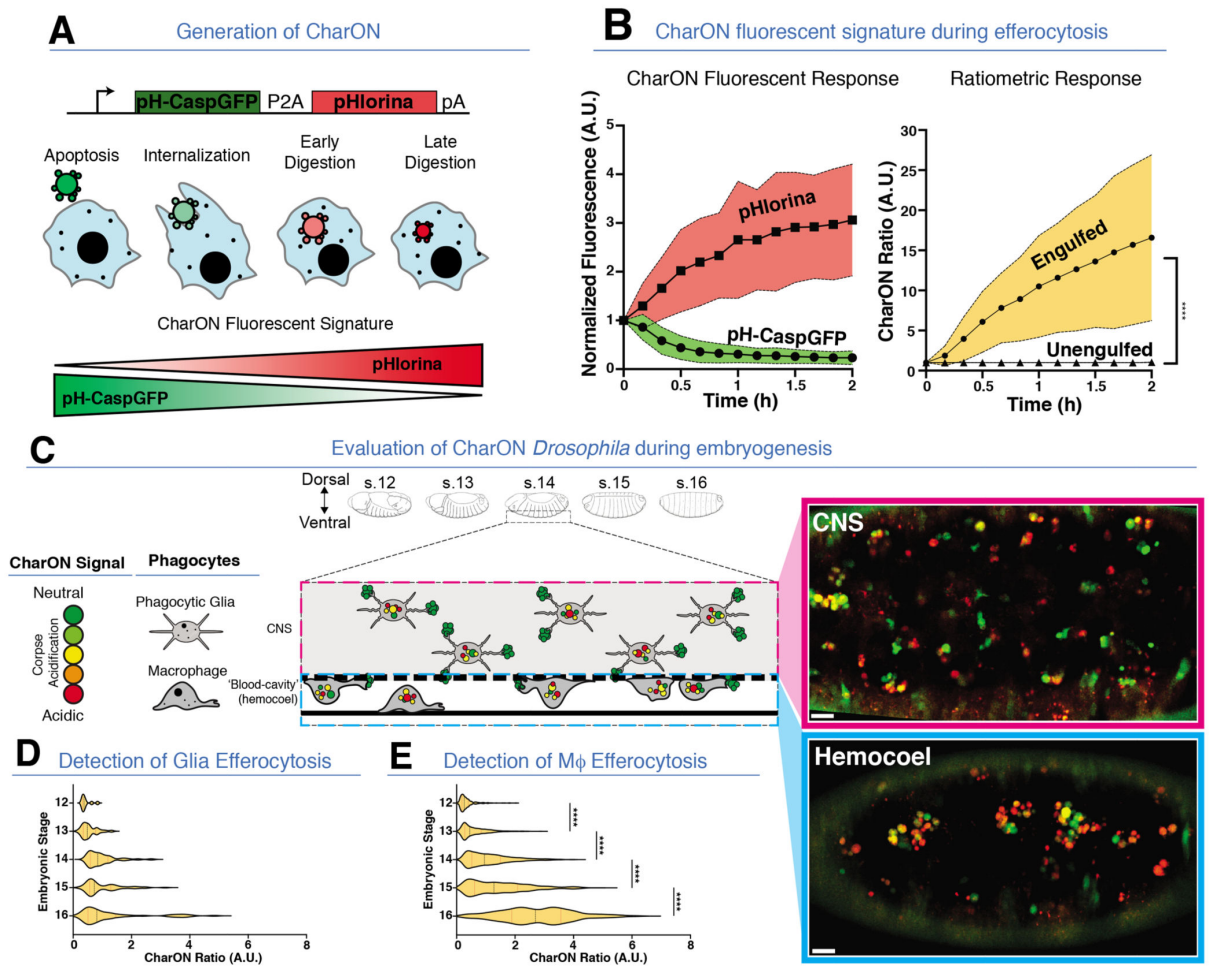
Caspase and pH Activated Reporter, Fluorescence ON (CharON) detects apoptosis and efferocytosis in vitro and in vivo. **(A)** CharON design and rationale. (Top) CharON construct design. (Bottom) Fluorescence of an apoptotic CharON expressing cell (green/red) engulfed by a macrophage (blue). **(B)** CharON fluorescence during efferocytosis. (Left) CharON pH-CaspGFP and pHlorina fluorescence during engulfment. (Right) Ratiometric CharON (pHlorina/pH-CaspGFP) signal during engulfment. Three independent experiments, Two-Way ANOVA, Šidák’s multiple comparison test, **** =p<0.0001. **(C)** (Left) CharON activity during *Drosophila* embryogenesis. Embryo outlines highlight morphological changes during mid-late embryogenesis (stages 12-16) (*36*). Extensive apoptosis occurs within the developing CNS (dashed box), which is cleared by phagocytic glia (pink dashed box). The ventral-most corpses at the interface between the CNS and the underlying ‘blood-cavity’ (hemocoel) are cleared by the macrophages (blue dashed box). (Right) CharON visualises efferocytosis within (top) the CNS (pink box) and (bottom) the hemocoel of a stage 14 embryo. **(D-E)** Efferocytosis increases during CNS development. CharON ratio (pHlorina signal/pH-CaspGFP signal) of individual corpses within CNS **(D)** and hemocoel **(E)**, during embryogenesis (stages 12-16). Five embryos/stage, One-Way ANOVA, **** =p<0.0001. All scale bars =10 μm.

**Fig. 3:**
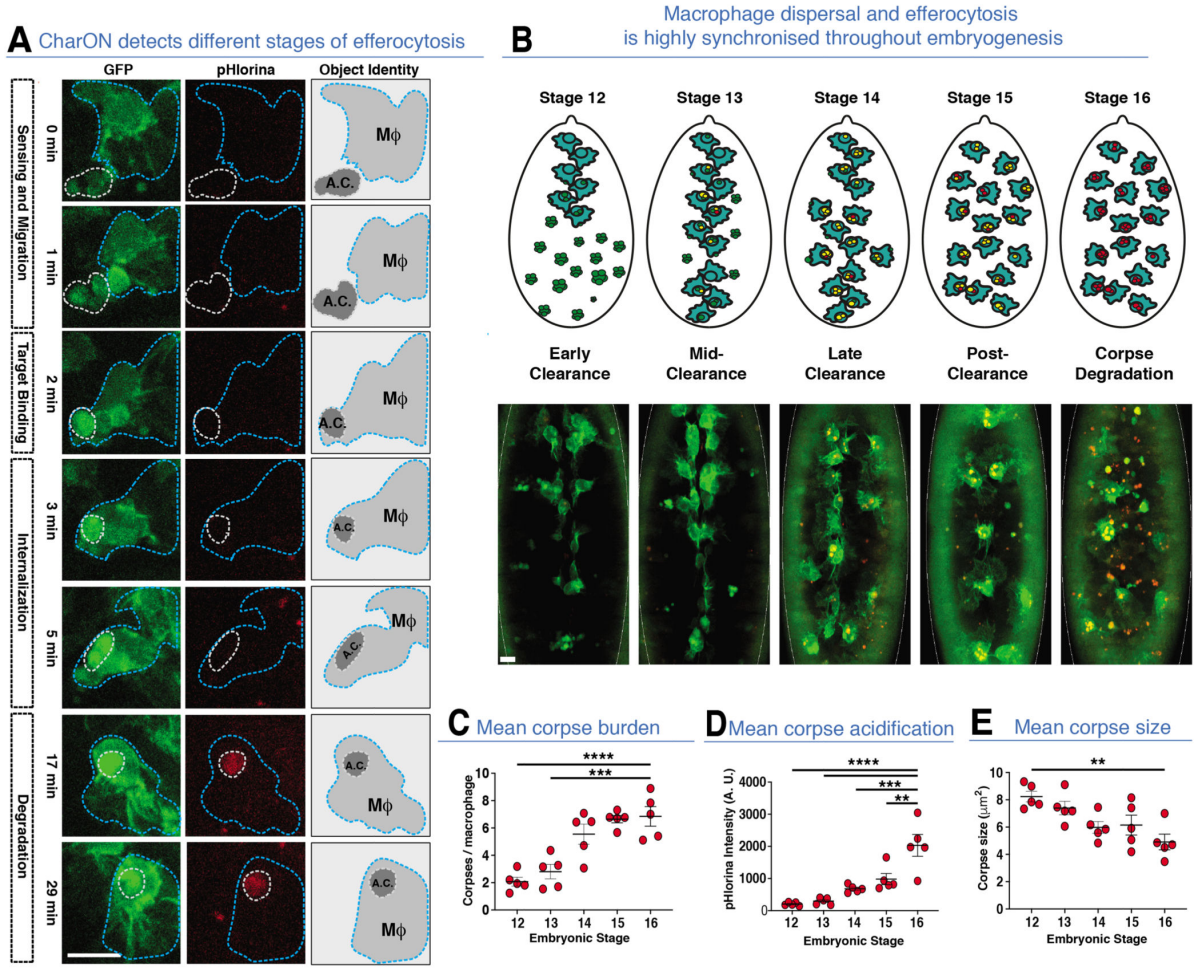
CharON illuminates macrophage-mediated efferocytosis in vivo. **(A)** CharON visualises the different stages of efferocytosis. A GFP-labelled macrophage (MΦ, blue dashed outline) engulfs a CharON-labelled apoptotic corpse (A.C., dashed outline) within the *Drosophila* embryo (stage 12). Apoptosis induces pH-CaspGFP (green) activation and macrophage (green) attraction, leading to target binding and uptake. Following internalisation, acidification/degradation of the corpse is detected through increasing pHlorina signal (red). **(B)** Efferocytosis increases during macrophage dispersal. (Top) Diagrams and (bottom) images highlighting the ventral dispersal of *Drosophila* macrophages (GFP, green) within the embryo (stages 12-16). During their stereotyped dispersal, macrophages clear CharON-labelled apoptotic corpses (green/red). The additional pHlorina-positive corpses are within unlabeled phagocytic glia deeper within the CNS. **(C-E)** Mean corpse number/macrophage (burden, **C**), pHlorina intensity/macrophage (acidification, **D**) and corpse area/macrophage (size, **E**) across stages 12-16 (embryo averages, 5 embryos/stage). One-Way ANOVA, ** =p<0.0021, *** =p<0.0002, **** =p<0.0001, error bars =S.E.M. All scale bar =10 μm.

**Fig. 4:**
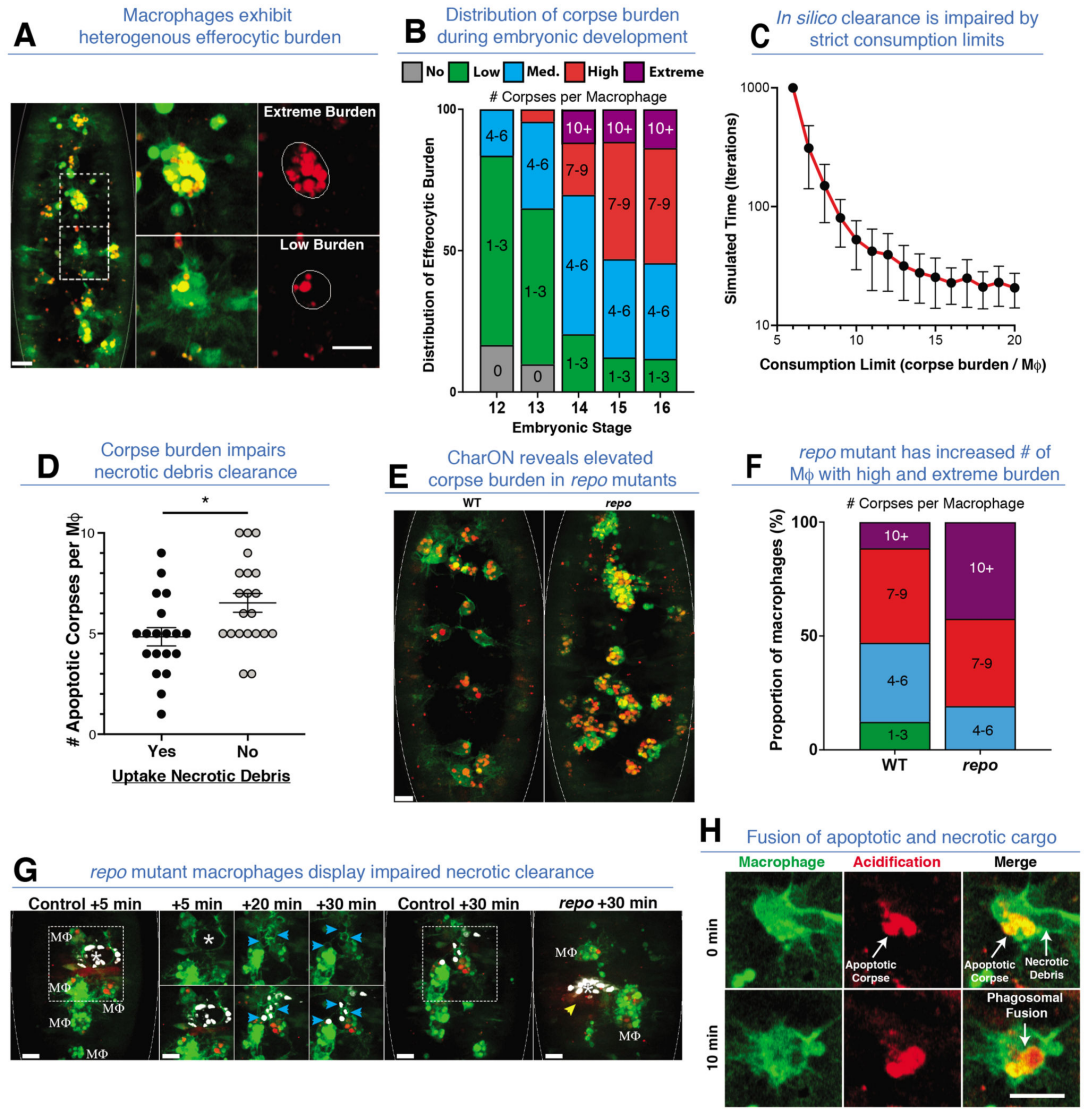
Macrophage efferocytic heterogeneity. **(A)** Macrophages exhibit variable corpse burden. (Left) A stage 16 embryo with GFP-labelled macrophages (green) and CharON-labelled apoptotic corpses (green/red). Two adjacent macrophages with contrasting corpse burdens are highlighted (dashed-boxes), magnified and outlined (middle and right). **(B)** Percentage of macrophages with indicated corpse burdens during embryogenesis (stages 12-16, 5 embryos/stage). **(C**) In silico clearance time is exponentially increased when equal sharing of corpses is enforced through consumption limits. Simulations run until clearance was completed or 1,000 iterations max and repeated 50x to yield standard deviations (error bars). (**D**) Macrophages with higher developmental apoptotic corpse burdens are significantly less likely to engulf necrotic debris at wounds (unpaired t-test, * =p<0.0332, 5 wounded embryos, error bars =S.E.M.). (**E-F**) Macrophages in *repo* mutants have elevated corpse burdens. **(E)** Wild-type (WT) and *repo* mutant stage 15 embryos (outlined) expressing CharON (green/red) and macrophage-specific GFP (green). **(F)** Percentage of macrophages with indicated corpse burdens in wild-type (WT) or *repo* mutant embryos (stage 15, 5 embryos/genotype). **(G)** *repo* mutant macrophages display impaired necrotic debris clearance. A necrotic stain (DRAQ7, white) was injected into control or *repo* mutant embryos (stage 15) expressing CharON (green/red) and macrophage (MΦ) specific GFP (green). Following laser-wounding (*), control macrophages cleared necrotic debris within 30 mins (blue arrows). In contrast, *repo* macrophages with extreme corpse burden failed to clear necrotic debris (yellow arrow). **(H)** Fusion of phagosomes containing apoptotic and necrotic cargo. A GFP-labelled macrophage (green) containing CharON-labelled apoptotic corpses (green/red) engulfs necrotic debris at a wound. Acidification of necrotic corpse was detected via pHlorina, occurring rapidly after interaction with an acidified apoptotic corpse. All scale bars =10 μm.

