# Supplementary material for: Live cell tracking of macrophage efferocytosis during Drosophila embryo development in vivo
Source: Science. Author manuscript; Available in PMC 2022 Mar 25. (PMC7612538; doi:10.1126/science.abl4430)
Supplement: Supplementary Data [file EMS143846-supplement-Supplementary_Data.docx]

Supplemental Materials for

**Live cell tracking of macrophage efferocytosis during Drosophila embryo development in vivo**

**The File Includes:**

Materials and Methods

Figures S1 to S10

Video Legends

References

**Materials and Methods**

Apoptosis Reporter Evaluation

To evaluate GFP-based apoptosis probes, we stably expressed GC3ai, FlipGFP and ZipGFP in Jurkat cells. The appearance of the caspase-induced green fluorescence signal of GC3ai aligned with phosphatidylserine exposure on the apoptotic cells, as measured by Annexin-V (Figures S1A, S1B, and S1C). When pH-CaspGFP (GC3ai with pH-stabilizing mutation Q204H) was stably expressed in Jurkat cells, apoptosis could be faithfully detected after different apoptotic stimuli, including ultraviolet-C irradiation and after treatment with ABT-737 (a BCL-2 inhibitor) or Staurosporine (Figure S2A). Furthermore, GFP+ apoptotic events scored via pH-CaspGFP fully matched the Annexin V^+^ events, and this was corroborated at different time points after death induction (Figures S1E and S1G). When pH-CaspGFP expressing cells were treated with the pan-caspase inhibitor Q-VD, the apoptosis/caspase-dependent GFP+ signal was reduced (Figure S2A), confirming caspase dependency. pH-CaspGFP was also compatible with detection of apoptosis induction in multiple cell types, including T cells (Jurkat cells), fibroblasts (LR73 cells) (Figure S2B) and monocytes (THP-1 cells) (Figures S2C and S2D).

CharON Construction:

For construction of CharON, we combined both pH-CaspGFP and pHlorina in a single transgene, driven by a single promoter. To genetically express both pH‑CaspGFP and pHlorina reporters in tandem, we split these fluorescent elements by a P2A self-cleaving site for equimolar expression (*12*).

*In vitro* cell death assays

Jurkat cells (ATCC) were treated with 150mJ of UV-C in a StrataLinker 1800 or 10 μM of ABT-737 (Abcam ab141336) in RPMI 1640 supplemented with 10% FBS and 1% Pen Strep with Glutamine. Cells were collected at indicated time points, stained 1:50 with Pacific Blue Annexin V (Biolegend #640917) and collected on an Attune Flow Cytometer (ThermoFisher). For each sample, 10,000 gated events were recorded, and data was processed in FlowJo V10. For each sample, singlet gates were used to distinguish single cells from doublets, followed by sub-gating on constitutively expressed RFP for analysis of GFP+ and Annexin V+ events. All RFP+, GFP+ and Annexin V+ event gates were set against the respective negative controls.

*In vitro* live cell imaging engulfment assays

UV-induced apoptotic CharON expressing Jurkat cells were co-cultured with mouse J774 macrophages (ATCC TIB-67) at 1:2 apoptotic cell:phagocyte ratios in 24 well plates and spun at 500rpm for 1 minute. Images were collected every 10 minutes for 24 hours on a Nikon Eclipse Ti2 scope using 40x magnification and kept under 37^o^C and 4% CO2 for the duration of the experiment. Raw Tiff images were exported for analysis using Fiji software.

Engulfment events were manually curated through identification of apoptotic pH-GC3ai (GFP+) cell internalization by J774 macrophages using co-localization between the apoptotic cell and the macrophage through brightfield. Upon identification of engulfment events, manually drawn circular regions of interest (ROI) were placed over identified events and tracked throughout the duration of the experiment. Intensity of phagocytic events were calculated using background subtracted GFP and RFP values.

Plasmid construction

In brief, all CharON constructs were inserted into a lentivirus backbone for expression in Jurkat cells. Gibson assembly PCR reactions using oligonucleotides (Integrated DNA Technologies) with 15bp overhangs were used to amplify and insert transgenes into restriction sites downstream of a SFFV promoter with an upstream CBX3 UCOE (UCOE-SFFV). In addition, P2A or T2A elements were used downstream of pH-GC3ai for bi-cistronic expression of mCherry or pHlorina. Sanger sequencing was used to verify sequences of inserted transgenes.

For construction of pH-CaspGFP, GC3ai was amplified from plasmid pCDH-puro-CMV-GC3AI (Addgene #78910). Mutation of GC3ai Q204H was performed via overlapping PCR and inserted into lentivirus UCOE-SFFV. CharON was constructed using pH-CaspGFP and pHlorina separated by a P2A site for bi-cistronic expression.

Lentivirus production, purification, and transduction

All lentivirus production was performed using Lenti-X HEK293T (Takara) grown in DMEM supplemented with 10% FBS and 1% Pen Strep with Glutamine. Cells were transfected at 80% confluency in 6-well plates with 2 ug total of psPAX2 packaging (Addgene #12260), pMD2.G envelope (Addgene #12259) and transfer plasmids at a 4:1:4 ratio. Supernatants were collected 48h after transfection and concentrated following the Lenti-X Concentrator (Takara) protocol.

Jurkat cells were plated in 24-well plates at 200k/well in RPMI 1640 supplemented with 10% FBS, 1% Penn Strep with Glutamine and 8µg/mL Polybrene. Purified viral supernatants were added to the wells and spun at 800g for 45min at 32 °C. After 24h, the cells were pelleted, washed in PBS and cultured in RPMI 1640 supplemented with 10% FBS and 1% Penn Strep with Glutamine. Transgene expression was assessed 72 hrs after transduction.

siRNA knockdown of Rubicon

For siRNA knockdown of *Rubicon*, 25nM of control or *Rubicon* targeting ON-TARGETplus SMARTpool siRNAs (Dharmacon) were transfected into mouse J774 macrophages using *Trans*IT-TKO per manufacturer’s instruction (Mirus) 48 hours before the efferocytosis assay.

siRNAs against *Rubicon*:

5’---CAGUUCAGUUCACGGGAUU---3’

5’---AUAUCAAGCGGCUGCGGUA---3’

5’---UCAGUUAACUAUCGUGAGU---3’

5’---GCACAGGAGAUGAGCGACA---3’

qPCR validation of siRNA knockdown

To validate qPCR knockdown of *Rubicon*, control or *Rubicon* targeting siRNA-treated J774s were harvested for RNA extraction after 48 hours of siRNA treatment. Total RNA was extracted from cells using the Rneasy Mini Kit (Qiagen) according to manufacturers’ instructions. cDNA was synthesized from RNA using the Quantitect Reverse Transcription Kit (Qiagen) according to manufacturers’ instructions. Quantitative gene expression of *Gapdh* and *Rubicon* was performed using mouse-specific Taqman probes (Applied Biosystems) run on a StepOnePlus RT-PCR System (Applied Biosystems).

General pHlorina genetic construction methods and materials

All synthetic DNA oligonucleotides for cloning and library construction were purchased from Integrated DNA Technologies (IDT). Taq DNA polymerase (New England Biolabs) was used for error-prone PCR (EP-PCR). PCR products and products of restriction digests were purified using gel extraction kit (BioBasic) according to the manufacturer’s protocols. Restriction enzymes and ligases were purchased from New England Biolabs or Thermo Scientific. The DNA sequences were analyzed at the University of Alberta Molecular Biology Service Unit (MBSU).

pHlorina protein engineering

Using mApple as a template, we created a library in which residues 161 and 163, both in close proximity of the chromophore, were randomized (*11*) (**Figure S3A**). Screening of this library led to the identification of mApple *Ile161Cys*/*Lys163Glu* with a large ratiometric pH response and high brightness. Further engineering via site-directed and random mutagenesis ultimately yielded a high-performance ratiometric pH sensor, which we designated pHlorina (**pH**-sensitive f**l**u**o**rescent **r**atiometric **in**dicator derived from m**A**pple, and pronounced ‘*florina*’) (**Figure S3A**).

Engineering of pHlorina was carried out by site directed mutagenesis and multiple rounds of Error-prone PCR (EP-PCR) using plasmids encoding mApple as a template. All synthetic DNA oligonucleotides for cloning and library construction were purchased from Integrated DNA Technologies (IDT). Taq DNA polymerase (New England Biolabs) was used for EP-PCR. PCR products and products of restriction digests were purified using gel extraction kit (BioBasic) according to the manufacturer’s protocols. Restriction enzymes and ligases were purchased from New England Biolabs or Thermo Scientific. The DNA sequences were analyzed at the University of Alberta Molecular Biology Service Unit (MBSU). All site-directed mutagenesis was performed using the Quikchange lightning mutagenesis kit (Agilent) and primers designed according to the manufacturer’s guidelines. EP-PCR products were digested with XhoI and HindIII and ligated into pBAD/His B vector digested with the same two enzymes and used to transform electrocompetent *Escherichia coli* strain DH10B (Thermo Fisher Scientific), which were then plated on agar plates containing LB medium supplemented with 0.4 mg/ml ampicillin and 0.02% w/v L-arabinose. Single colonies were picked and inoculated into 4 ml of LB medium with 0.1 mg/ml ampicillin and 0.02% w/v L-arabinose and then cultured overnight. Protein was extracted using B-PER bacterial extraction reagent (Thermo Fisher Scientific) as per manufacturer guidelines. Screening for pH sensitivity of extracted proteins was performed with a Safire2 fluorescence plate reader (Tecan) by measuring protein fluorescence excitation and emission spectra in buffers of pH 5.0 and 7.5. Plasmids were purified with the DNA miniprep kit (Thermo Fisher Scientific) and then sequenced using the BigDye Terminator Cycle Sequencing kit (Applied Biosystems). We combined approaches of rational site-directed mutagenesis and random mutagenesis to improve the pH-induced fluorescence change. Based on pHluorina0.1 (mApple Ile161Cys/Lys163Glu), we first randomized position Ala217, the corresponding residue of a crucial mutation in pHRed, which rendered a larger ratio change compared to its template mKeima. The Ala217X (X = all 20 common amino acids) mutant library was expressed and screened by measuring the excitation spectra at pH 5.5 and 7.5. One variant with the largest excitation ratio change was selected and designated as pHlorina0.2. Sequencing revealed pHlorina0.2 had a mutation of A217T, Which is similar to the A217S mutation in pHRed (*35*). To increase the brightness of the pHlorina0.2, another saturation mutagenesis was performed at the position 143, The brightest variant in this 143X library, pHlorina0.3, possessed a W143Y mutation. Multiple rounds of random mutagenesis and screening for maximum red fluorescence increase at acidic pH were performed based on pHlorina0.3, eventually led to pHlorina.

Imaging of pHlorina in cell culture for calibration of pH dependency:

HeLa cells were grown in DMEM supplemented with 10% fetal calf serum, 1% sodium pyruvate, and 1% Glutamax with 1% penicillin/streptomycin and maintained at 37°C in 5% CO_2_. pDisplay-pHlorina was made by subcloning pHlorina into the pDisplay vector (Life Technologies), between the BglII and SalI restriction sites. The pDisplay vector is translated into a protein consisting of the N-terminal signal peptide, pHlorina, and a C-terminal transmembrane domain of platelet-derived growth factor receptor (PDGFR) which enables anchoring of the fused protein to the cell surface, hence its sensitivity to changes in extracellular pH. Twenty-four hours before imaging, cells were transfected with 2 µg of plasmid DNA and 6 µl of X-TREMEGene-HP (Roche), mixed in PBS for 10 min and added directly in the culture medium. 4 h later, 30000 cells were plated on 18-mm glass coverslips. Live cell imaging was done at 37°C. Cells were perfused with solution containing 135 mM NaCl, 5 mM KCl, 0.4 mM MgCl_2_, 1.8 mM CaCl_2_, 20 mM HEPES, and 1 mM D-glucose, adjusted to pH 7.4 and 315 mOsmol/L. For pH titration, solutions were prepared as the HBS solution described above using as pH buffers HEPES for solutions at pH 7-10, PIPES for solutions at pH 6 and 6.5 and MES for solutions at pH 5 and 5.5. TIRF imaging was performed on an inverted microscope (IX71; Olympus) equipped with an Apochromat N oil 60× objective (NA 1.49), a 1.6× magnifying lens, and an electron multiplying charge coupled device camera (QuantEM:512SC; Roper Scientific). Samples were illuminated by a 473-nm laser and a coaligned 561-nm laser. Emitted fluorescence was filtered using a 620/60 nm filter (Chroma Technology Corp.) for pHlorina imaging. In situ pH titration revealed pDisplay-pHlorina has a p*K*_a_ of 6.2 and n_H_ of 1.6, with a maximum ΔF/F_0_ of 2.6 (**Figure S3B**). During imaging, we noted the presence of brightly fluorescent clusters which are not sensitive to change in extracellular pH, which is likely to be acidic intracellular organelles such as the lysosome. To verify this, we applied ammonium chloride (NH_4_Cl, 50 mM) to neutralize the luminal pH of intracellular organelles. This treatment cancelled the fluorescence of bright clusters, suggesting the fluorescence is from pHlorina signal in intracellular organelles (**Figure S3B**). Taking advantage of the ratiometric dual excitation, we also imaged the cells under 473 and 561 nm laser excitation, the bright fluorescent clusters were only visible with 561 nm excitation but not with 473 nm excitation (**Figure S3D**), further confirming that bright fluorescent clusters are from pHlorina in an acidic environment.

pHlorina *in vitro* characterization:

To purify the proteins, electrocompetent *E. coli* strain DH10B (Invitrogen) was transformed with the plasmid of interest using a Micropulser electroporator (Bio-Rad). Transformed bacteria were cultured overnight on agar plates containing LB and ampicillin. Single colonies were picked and grown overnight in 4 mL LB supplemented with ampicillin at 37 °C. For each colony, the 4 mL culture was then used to inoculate 250 mL of LB medium with ampicillin and grown to an optical density of 0.6. Protein expression was induced with the addition of 0.02% L-arabinose and the culture was grown overnight at 37 °C. Bacteria were harvested at 10,000 rpm, 4 °C for 10 min, lysed using a cell disruptor (Constant Systems) and then clarified at 14,000 rpm for 30 min. The protein was purified from the supernatant by Ni-NTA affinity chromatography (ABT) according to the manufacturer’s instructions. The buffer of the purified protein was exchanged with 10 mM Tris-Cl, 150 mM NaCl, pH 7.3 with Amicon ultra centrifugal filter (MWCO 10,000) for a final protein concentration of approximately 10 μM. Fluorescence intensity as a function of pH was determined by dispensing 2 μL of protein solution into 50 μL of the desired pH buffer in triplicate into a 384-well clear-bottom plate (Nunc) and measured in a Safire2 plate reader. pH Buffer solutions from pH = 3 to pH = 11 were prepared according to the Carmody buffer system (*32*).

Drosophila genetics including generation of CharON transgenics

CharON construct was cloned downstream of UAS sequence in pUASt-ATTB, which in turn was used to generate transgenic CharON flies. This was recombined with daugherless GAL4 (da-gal4) to drive ubiquitous expression throughout embryo (*;; da-gal4, uas-charon*) (*37*). Srp-GMA or srp-3xmCherry (*38*) were used for the GAL4-independent labelling of macrophages (*; srp-gma; da-gal4, uas-charon* or *; srp-3xmcherry/+ ; da-gal4, uas-charon*). The *repo^03702^* allele (amorphic null) (*24*) was recombined with *;;* *da-gal4, uas-charon* and combined with srp-GMA to yield *; srp-gma/CyOdfd ; repo^03702^, da-gal4, uas-charon*/TM6b-dfd flies, with the embryonic fluorescence conferred by the balancer chromosomes allowing the selection of homozygous embryos (*34*). Macrophages and phagocytic glia where co-labelled using *; sn-gal4,uas-lifeact-gfp* (*31*)*.*

Imaging of *Drosophila* embryos

Embryos laid on apple juice agar plates overnight were collected in cell strainers (Falcon), dechorionated with bleach (Jangro), and washed with purified water. Embryos at developmental stages 12-16 were mounted on double-sided tape on a glass slide in droplets of VOLTALEF oil (VWR), beneath a bridging coverslip (No. 1.0, SLS Ltd) sealed on top of two supporting coverslips (No. 1.5, SLS Ltd). Z-stacks of the ventral side of embryo were captured using a Zeiss LSM 880 (with Airyscan fast) confocal microscope using a plan-apochromat 40x objective with a NA of 1.3. Epithelial wounds were generated using laser ablation (nitrogen-pumped Micropoint ablation laser tuned to 435 nm, Andor Technologies) as previously described (*34*). The acquisition software used was Zen Black (Zeiss). Alternatively, a spinning disc confocal microscope (Perkin Elmer Ultraview) using a plan-apochromat 63x objective with a NA of 1.4 and a Hamamatsu C9100-14 camera, was used for long-term, live-imaging of macrophage dispersal. The acquisition software used was Volocity (Perkin Elmer). For 3-colour imaging of DRAQ7 labelled necrotic debris, stage 15 embryos were mounted as described above, but dehydrated in a desiccation box with silica beads for 20-30 minutes pre-VOLTALEF oil (VWR) application. After a droplet of oil was added to each embryo, 0.3 mM DRAQ7 (Thermo Fisher Scientific) was injected anteriorly into the intervitelline space. The injection was performed using a FemtoJet injectman rig (Eppendorf) fitted with Femto tips (Eppendorf). A coverslip was sealed on top before wounding and spinning disc confocal microscopy as described above.

In vivo image analysis

ImageJ (NIH) was used for all image analysis. All the images present in the figures and movies of this manuscript are z-projections and are adjusted only for contrast and brightness, with occasional use of the ‘despeckle’ tool in imageJ to adjust for noise (never used during quantification).

For quantification of efferocytosis across embryogenesis, individual 20 µm z-stacks of 5 embryos per developmental stage (25 total) were imaged using scanning confocal microscopy and quantified for each genotype. CharON labelled corpses that were superficial to the CNS were isolated by cropping in on a 5 µm z-stack that minimally contained a cluster of apoptotic bodies. These were then flattened (average intensity projection) and each apoptotic body was outlined manually using pH-CaspGFP signal. Apoptotic bodies deeper within the CNS were isolated by z-projecting (average intensity) the slices from the deepest 5 µm of the z-stack into the embryo. Apoptotic corpses were again outlined manually using pH-CaspGFP signal. For identification of apoptotic bodies specifically within ventral macrophages, 5 µm z-stacks that minimally contained the macrophages (guided by srp-GMA label) were isolated and z-projected (average intensity). CharON-labelled apoptotic corpse bodies were outlined manually using pHlorina signal (due to pH-CaspGFP overlap with srp-GMA). Area and mean intensities within these outlines were measured and their numbers counted.

For time-lapse imaging of macrophage efferocytosis, dispersing macrophages (embryonic stages 12-13) were imaged using spinning disc confocal microscopy (1 z-stack/minute). Engulfed apoptotic bodies with strong pH-CaspGFP signal (detectable above srp-GMA) were identified and a 2 µm z-stack that minimally contained the corpse was isolated (position of this z-stack adjusted in x, y, and z for every frame to ensure apoptotic body was contained centrally within z-stack as it moved around embryo within a macrophage). These z-stacks were flattened (maximum intensity projections) and the apoptotic body was outlined manually in each frame (starting from moment of uptake). Mean intensities within these outlines were then calculated for each frame. Following background subtraction, fold-change in intensity from initial value were calculated and plotted.

Macrophage speeds, chemotaxis (FMI) and distances travelled were calculated by manually tracking cell centroids using the ImageJ Manual tracking and chemotaxis tool plugins. For analysis of ventral macrophage basal migration and inflammatory chemotaxis in response to wounding, stage 15 embryos (5 unwounded and 5 wounded embryos respectively) were imaged using scanning confocal microscopy (1 z-stack/minute). The number of CharON-labelled apoptotic bodies within each macrophage was determined in the initial z-stack (as described above) and macrophages were then tracked, again using ImageJ Manual tracking and chemotaxis tool plugins. Responders to wounds were defined as macrophages that moved to the wound edge, wherein tracking was stopped. Non-responders represent cells that were never recruited to the wound edge and were tracked throughout the entire time-lapse. Necrotic debris uptake at wounds was observed as fluorescent-negative ‘vacuoles’/voids within the GFP-labelled macrophages. The uptake of such debris was scored for each macrophage for the entire time it was at the wound edge. Alternatively, the number of DRAQ-labelled corpses per wound recruited macrophage was counted (≥60 min post-wounding). Note DRAQ7 signal bleeds into red (i.e. pHlorina) channel.

To generate *Drosophila* heatmaps, processed GFP and RFP image z-stacks from each developmental stage were substacked into Hemocoel (1-30) and CNS (31-99) regions using Fiji software. Images were corrected for alignment using the Rotate feature before importing into Ilastik for object identification. Using Ilastik, a model was trained through manual annotation on GFP and RFP channel images in order to segment apoptotic and acidification events, respectively. Segmented images were used for object identification, followed by object masking for quantification of events. A total of four to five embryos per stage were used to evaluate the total number of events within the Hemocoel.

Methods for simulation of agent-based models

Agent-based models (ABMs) allow for the exploration of complex behaviour by applying a simple set of rules. We can use ABMs to represent our biological system by assuming that agents can be used to model macrophages and apoptotic cells. There are many ABM environments currently available, we chose to use the Scala/Java-based modelling suite NetLogo. NetLogo offers a robust amount of pre-programmed agent primitives which aid in model building and application. NetLogo also offers three types of agents: patches, turtles, and links. Patches are immovable agents, turtles are moveable agents and links are linking agents, each of these agent types have their own in-built primitives.

1. Phagocyte clearing model

The experimental burden per macrophage is well described by a binomial distribution (supplementary figure 8A) implying that the targets are randomly distributed amongst the macrophages. This motivated the use of a random walk to model macrophage migration, as this captures the behaviour of macrophages having an equal probability of coming across and consuming a fragment. To model phagocyte clearing we simulated a domain of a rectangular lattice with Lx = 50 and Ly = 25. Within NetLogo the lattice sites are referred to as patches which is the convention we will now follow. We model macrophages as moveable agents *(turtles),* and the apoptotic cells as immovable agents *(patches*).

To set up the simulation, macrophages were randomly distributed around the modelling domain, with the following volume exclusion rule: one macrophage per patch. The number of macrophages was fixed to 88 within the model which fits the experimental area number density (supplementary figure 8A). Apoptotic cells were then randomly distributed around the remaining domain with the following volume exclusion rules: one cell per patch and the patch cannot be already occupied by a macrophage. The number of apoptotic cells in the simulation was fixed to the experimental ratio of 1.1:1 apoptotic cell to macrophages. For simplicity periodic boundaries were applied at all boundaries.

To run the simulation, under these conditions, the macrophages check to see if there are any apoptotic cells within a sensing radius of 3 patches (supplementary figure 8B). The distance between patches is calculated as the Euclidean distance between patch centre to centre. If any apoptotic cells are present the macrophage preferentially jumps to the closest cell. If multiple macrophages choose the same cell, one of the macrophages moves to that cell and the others choose their second nearest cell. When no consumption limit is applied; within one iteration the macrophage eats a uniformly drawn random amount of corpse fragments available from the apoptotic cell. The amount eaten is set between 1 and the maximum number of fragments available. The macrophage then moves away from the cell by a maximum distance of 3.3 patches (equivalent to the 33 µm maximum distance observed in the experimental data), to give other macrophages the chance to move onto the cell and consume fragments. When a consumption limit is applied the macrophages undergo the same process, however, the maximum number of fragments available to consume is dependent on the consumption limit, i.e., if a macrophage has a consumption limit of 10 and they have already consumed 4 cell fragments, they can only consume a number between 1 and 6. If a macrophage reaches the consumption limit, they then only undergo a random walk. In the case where there are no apoptotic cells within a macrophage’s sensing radius, the macrophage undergoes a random walk where-in they choose one of their direct neighbours to move into with equal probability. Here we define the direct neighbours as the eight surrounding patches of the patch the macrophage is currently on. Simulations were run for 1000 iterations or until all apoptotic corpses were cleared from the domain, whichever happens first. Each simulation is then run 100 times with different random initial positions for the macrophage and apoptotic cells. The distribution of apoptotic cell fragments amongst the macrophages is then calculated along with the maximum time needed to clear the domain.

1. Chemotaxis model

The rules implemented within the agent-based model for chemotaxis are the same as the phagocyte model with adaptions made to how macrophages and apoptotic cells are created along with the motion of the macrophages when a linear chemotactic field is assumed.

To set up the simulation, macrophages were randomly distributed along the top two rows of the domain (Ly = Ly_max_ and Ly = Ly_max - 1_) with the following volume exclusion rules: one macrophage per patch. Apoptotic cells were then randomly distributed around the remaining domain with an exclusion applied to the rows; Ly = Ly_max_ and Ly = Ly_max – 1_. Periodic boundary conditions were applied to the Lx boundaries, but reflective boundaries were applied at the Ly boundaries. While running the simulation, if the macrophage has reached its consumption limit, then it will undergo a biased random walk until it reaches y = y_min_. In the case where there are no apoptotic cells within the macrophage sensing radius, the macrophages undergo a biased random walk where-in they choose one of their front. neighbours to move to with equal probability. Here we define the front neighbours as the 3 patches in-front of the patch the macrophage is currently on, I.e., y_front_ < y_current_. Simulations were run with the same number of iterations as the phagocyte clearing model. The distribution of apoptotic cell fragments amongst the macrophages is then calculated along with the apoptotic clearance %.

**Fig. S1**


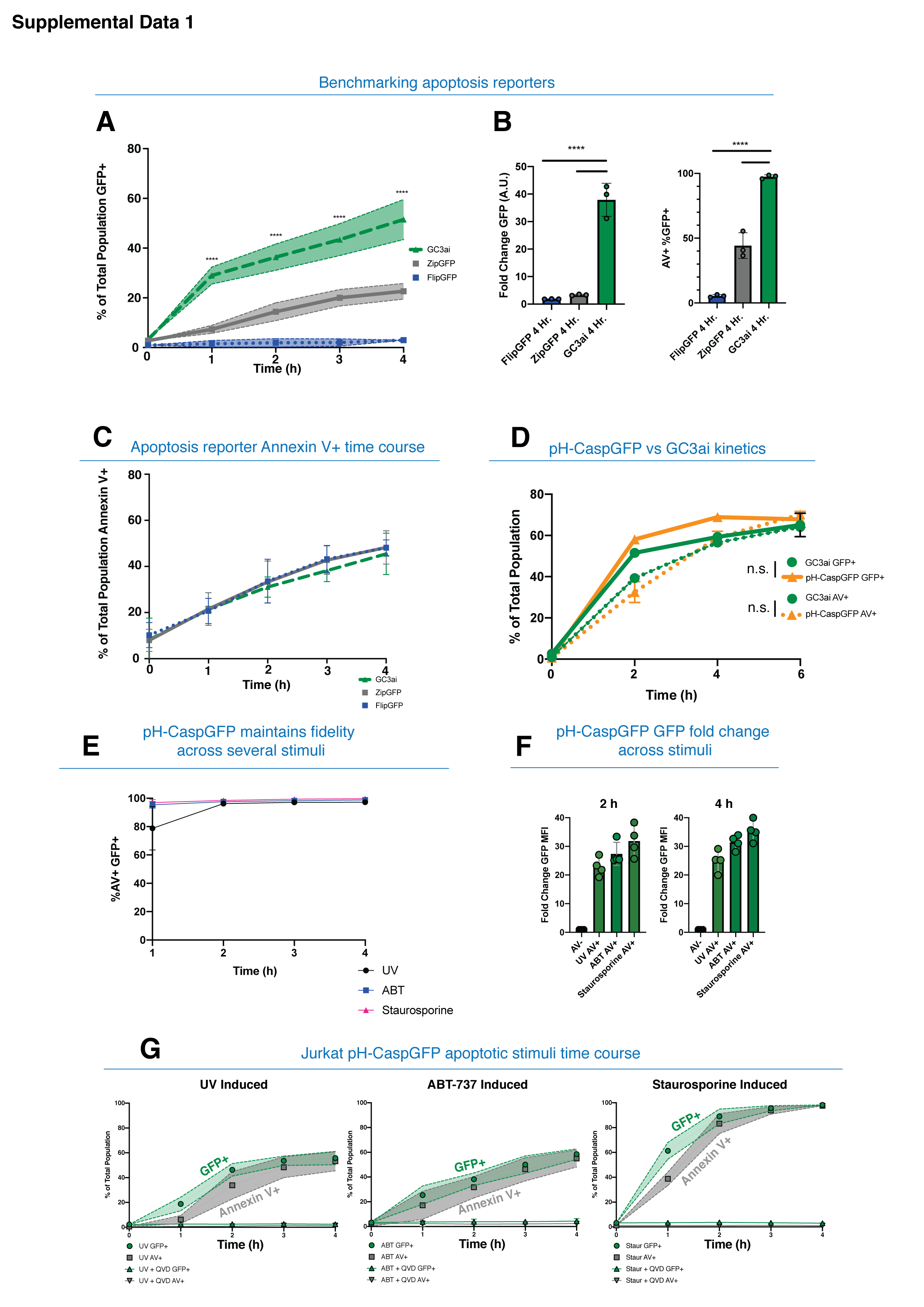


**Fig. S1: Benchmarking apoptosis reporters and pH-CaspGFP.** (**A**) Jurkat cells stably expressing GC3ai, ZipGFP and FlipGFP were subjected to 150mJ UV-C and monitored over 1-hour intervals for the appearance of GFP+ events via flow cytometry. N=3 independent experiments. (Two-Way ANOVA, **** =p<.0001). (**B**) (left) Fold changes in fluorescence of GC3ai, ZipGFP, and FlipGFP expressing Jurkat cells were compared after 4 hours of UV-C exposure. GFP Fold Change was calculated compared to T=0 hr. (One-Way ANOVA, **** =p<.0001) (right) Fidelity of apoptosis detection of GC3ai, ZipGFP and FlipGFP stably expressing Jurkat cells were determined through proportion of double-positive (Annexin V+ / GFP+) events. N=3 independent experiments. (One-Way ANOVA, **** =p<.0001). (**C**) Phosphatidylserine on apoptotic cells (measured via Annexin V) on GC3ai (green triangle), ZipGFP (gray square) and FlipGFP (blue square) expressing Jurkat cells upon UV-C exposure over a time course. (**D**) pH-CaspGFP (orange) and GC3ai (green) GFP+ and Annexin V+ signals were determined over a time course upon UV-C exposure. N=3 independent experiments. (One-Way ANOVA, n.s. = not significant). (**E**) pH-CaspGFP stably expressing Jurkat cells were subjected to either UV-C (black circle), ABT-737 (blue square) or Staurosporine (magenta triangle) and monitored over 4 hours for percentage of Annexin V+ events which are GFP+ (Annexin-V+ / GFP+) events. (**F**) GFP^+^ Fold Change in pH-CaspGFP stably expressing Jurkat cells after 2 and 4 hours post-exposure to UV-C, ABT-737 or Staurosporine, compared to T=0 hr. (**G**) Time-course of Annexin V+ (gray square) and GFP+ (green circle) events upon exposure of pH-CaspGFP stably expressing Jurkat cells to UV-C, ABT-737 or Staurosporine. In addition, cells pretreated with caspase inhibitor Q-VD was used as a control (triangle).

**Fig. S2**


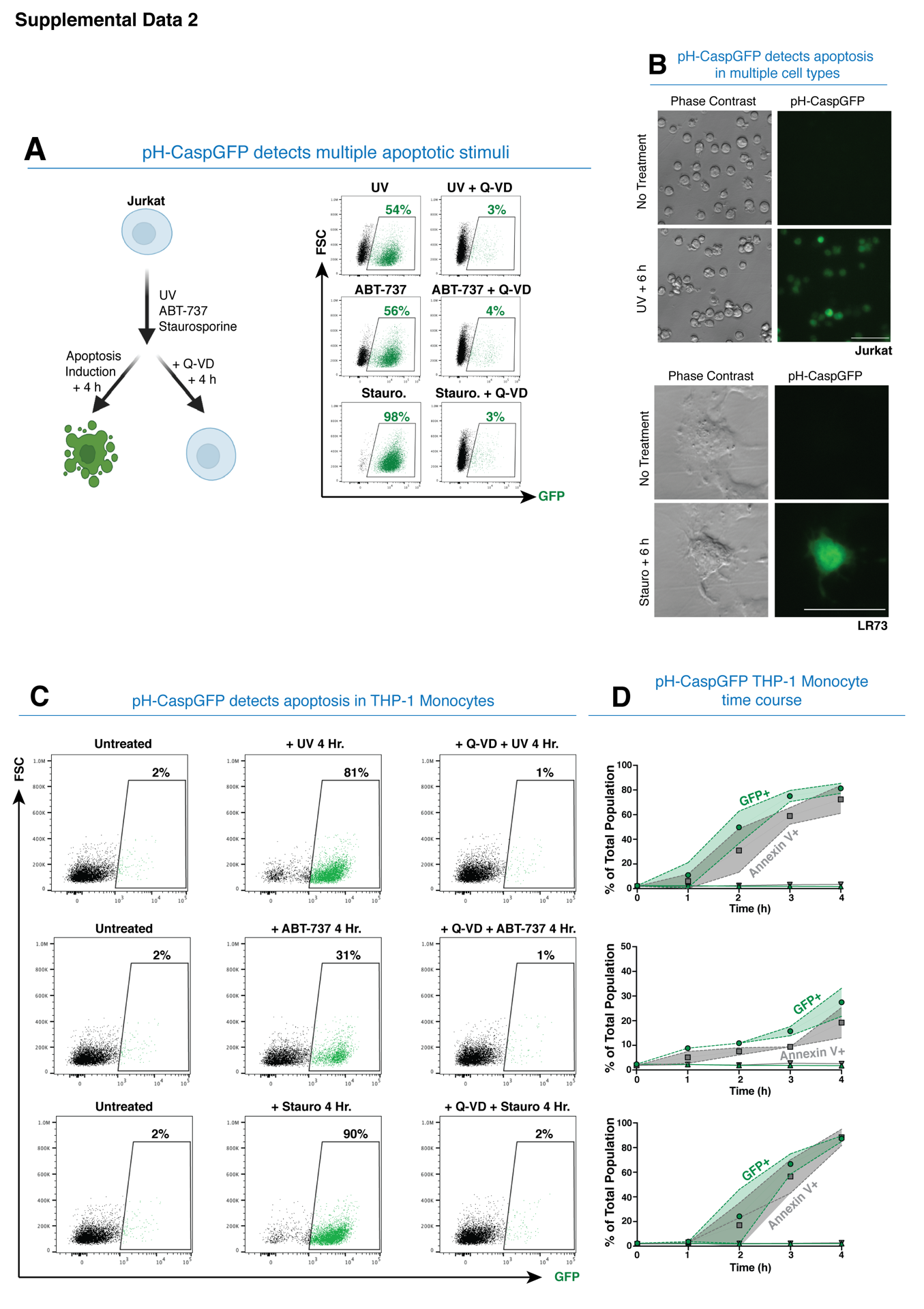


**Fig. S2: pH-CaspGFP responds to multiple stimuli and works in multiple cell lines.** (**A**) pH-CaspGFP expressing Jurkat cells were killed with UV-C, 20nM ABT-737 and 1µM staurosporine and measured for GFP+ events over four hours. To block apoptosis (and test the fidelity of pH-CaspGFP), Jurkat cells were pre-treated with 50 µM Q-VD. (**B**) Stably pH-CaspGFP expressing Jurkat cells and LR73 cells were rendered apoptotic via UV and 1µM staurosporine, respectively. GFP+ cells were assessed via time-lapse imaging. Scale bars =50 µm. (**C**) THP-1 cells stably expressing pH-CaspGFP were treated with UV-C (top row), ABT-737 (middle row), or Staurosporine (bottom row) and analyzed after 4 hours for presence of GFP+ events. Cells pretreated with caspase inhibitor Q-VD were used as a control. (**D**) Time-course of THP-1 cells stably expressing pH-CaspGFP after treatment with UV-C (top), ABT-737 (middle) or Staurosporine (bottom) and collection of GFP+ (green circle) and Annexin V+ events (gray square, indicative of phosphatidylserine flipping during apoptosis). Cells pretreated with caspase inhibitor Q-VD was used as a control (triangle).

**Fig. S3**


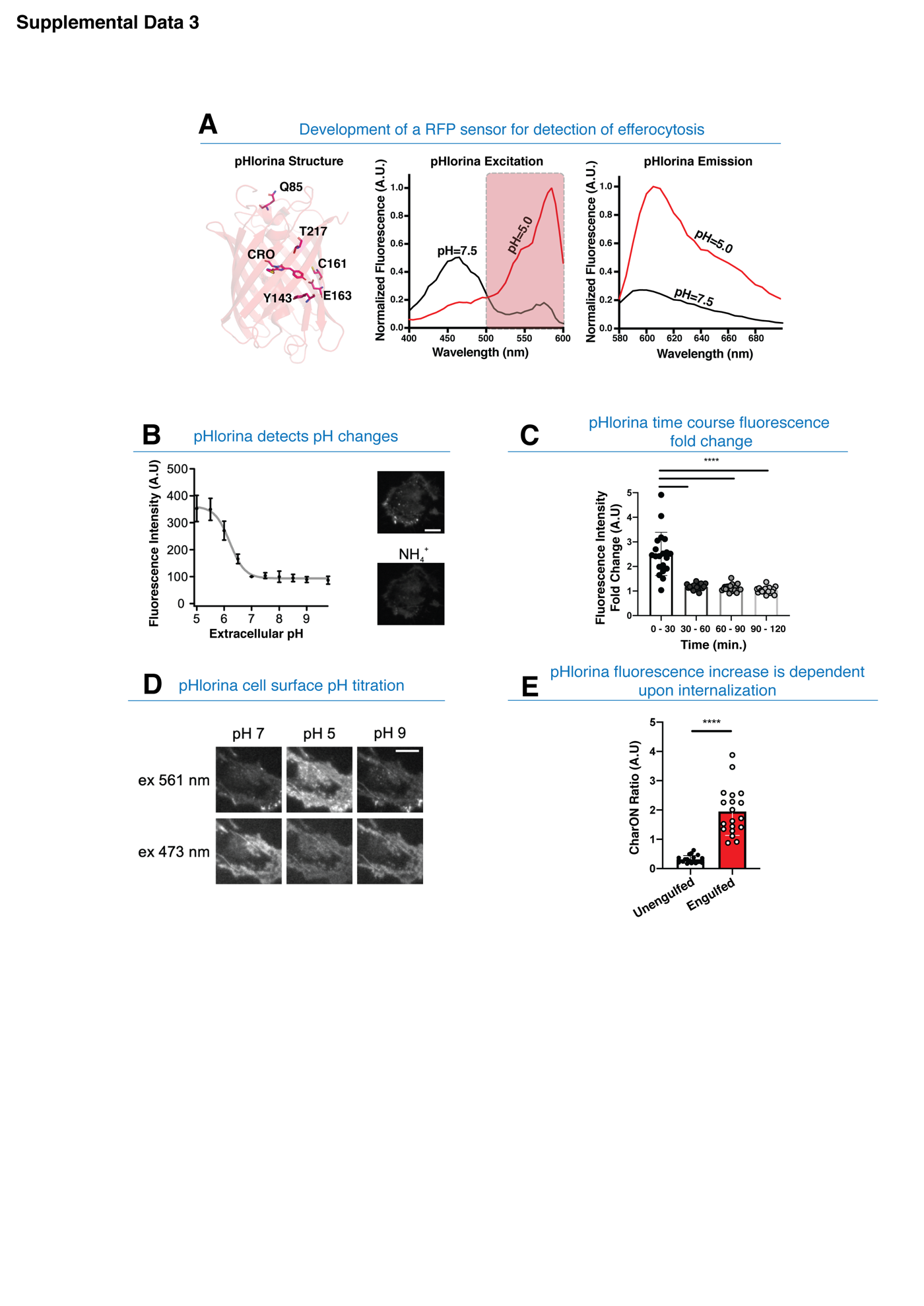


**Fig. S3: Generation and evaluation of pHlorina.** (**A**) Left: Modeled structural representation of pHlorina and mutations relative to the parental RFP mApple (based on mCherry structure, PDB 2H5Q). (middle) Excitation spectra of purified pHlorina protein from 400 nm to 600 nm at pH 5.0 (red) and pH 7.5 (blue). Right: Emission spectra of purified pHlorina with 560 nm excitation at pH 5.0 (red) and pH 7.5 (blue). (**B**) Left: Quantification of cell fluorescence intensity at pH values ranging from pH 5 to 9.8 (n=5 cells), with pH 7 values normalised to 100. Right: Representative image of HeLa cells transfected with pDisplay-pHlorina before and after the application of a solution at pH 7, containing 50 mM NH_4_Cl. (**C**) Apoptotic cell-derived fold change fluorescence change of pHlorina after internalization by macrophages binned into 30 minute intervals. Fluorescence intensity fold change was compared to T= 0 min. N=3 independent experiments. (Two-Way ANOVA, **** = p<.0001). (**D**) Representative images of HeLa cells transfected with pDisplay-pHlorina, with fluorescence excited with 561 nm or 473 nm Laser at extracellular pH of 5, 7, and 9. (**E**) Unengulfed and engulfed CharON expressing Jurkat cells were compared during in vitro efferocytosis assays to determine fluorescence response. N=4 independent experiments. (Unpaired T-Test, **** = p<.0001).

**Fig. S4**


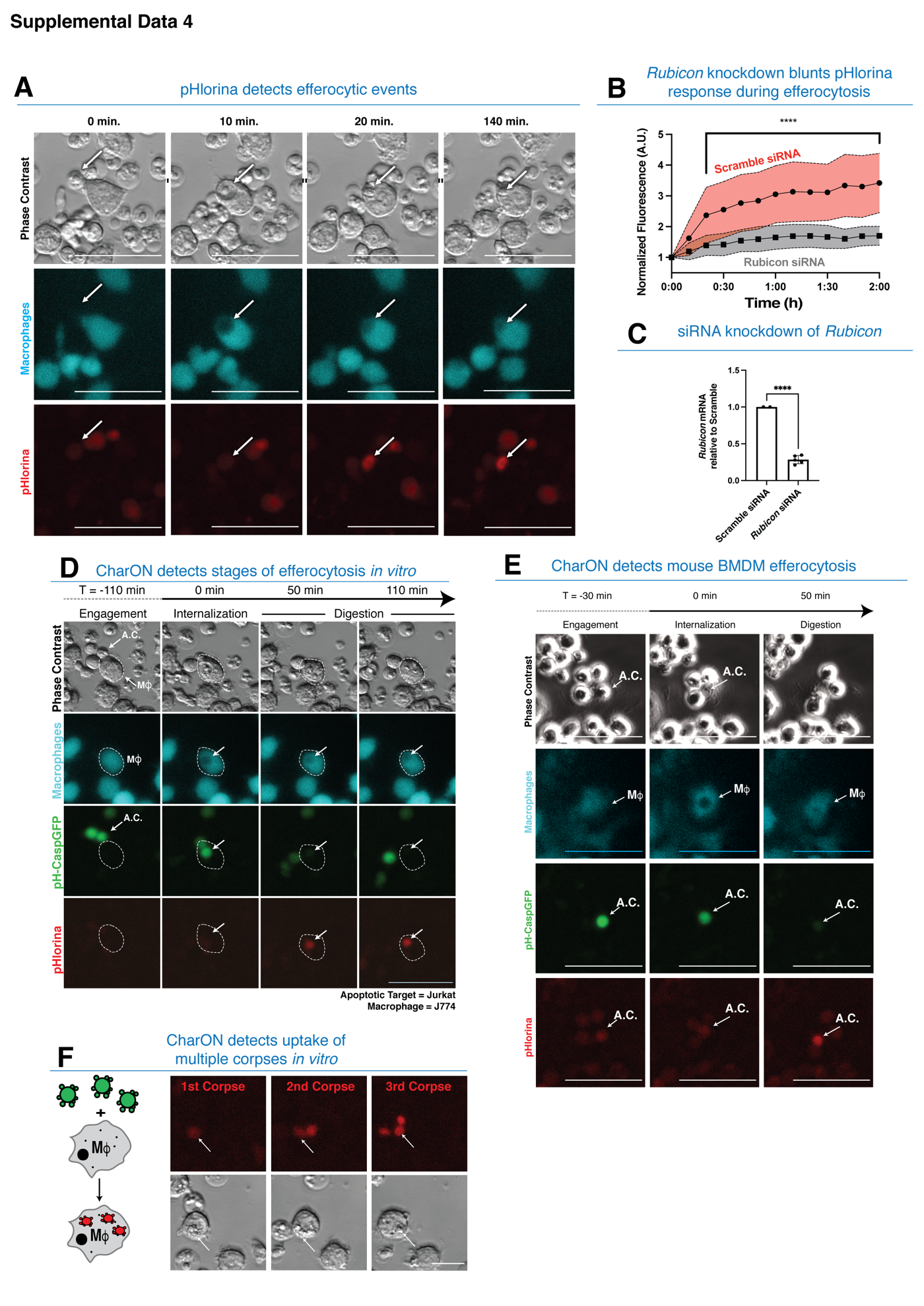


**Fig. S4: CharON detects efferocytosis in vitro.** (**A**) Mouse J774 Macrophages (cyan) were co-cultured with apoptotic pHlorina expressing Jurkat cells (red) during *in vitro* engulfment assays. Arrows indicate engulfment of pHlorina expressing Jurkat cells by mouse J774 macrophages starting at T = 0 min. Scale bars = 50 µm. (**B**) siRNA mediated knockdown of *Rubicon* in J774 macrophages blunts pHlorina fluorescence increase during efferocytosis of apoptotic pHlorina Jurkat cells. N=2 independent experiments. Two-way ANOVA, **** = p<.0001. (**C**) J774 macrophages were treated with 25nM scramble or *Rubicon* targeting siRNA for 48h. Each sample was normalized to *Gapdh* expression. Scramble siRNA n=2, *Rubicon* siRNA n=5 Unpaired t-test, **** = p<.0001. (**D**) J774 macrophages were incubated with apoptotic CharON expressing Jurkat cells to detect efferocytic events. CharON GFP and RFP signal was evaluated via Time-Lapse imaging during the efferocytic process. A.C. = Apoptotic Cell, Mɸ = Macrophage. Scale bars =50 µm. (**E**) Mouse HoxB8 bone marrow derived macrophages were co-cultured with apoptotic CharON expressing Jurkat cells (A.C.) during *in vitro* engulfment assays. Arrows indicate engulfment of CharON expressing Jurkat cells by mouse Hoxb8 bone marrow derived macrophages starting at T = 0 min. Scale bars = 50 µm. (**F**) (left) Schematic of *in vitro* engulfment of multiple CharON corpses by a macrophage over 2 hours. (right) Images of successive engulfment events by a single macrophage; arrows indicate internalized and acidified corpses revealed by the pHlorina signal. Scale bars = 20 µm.

**Fig. S5**


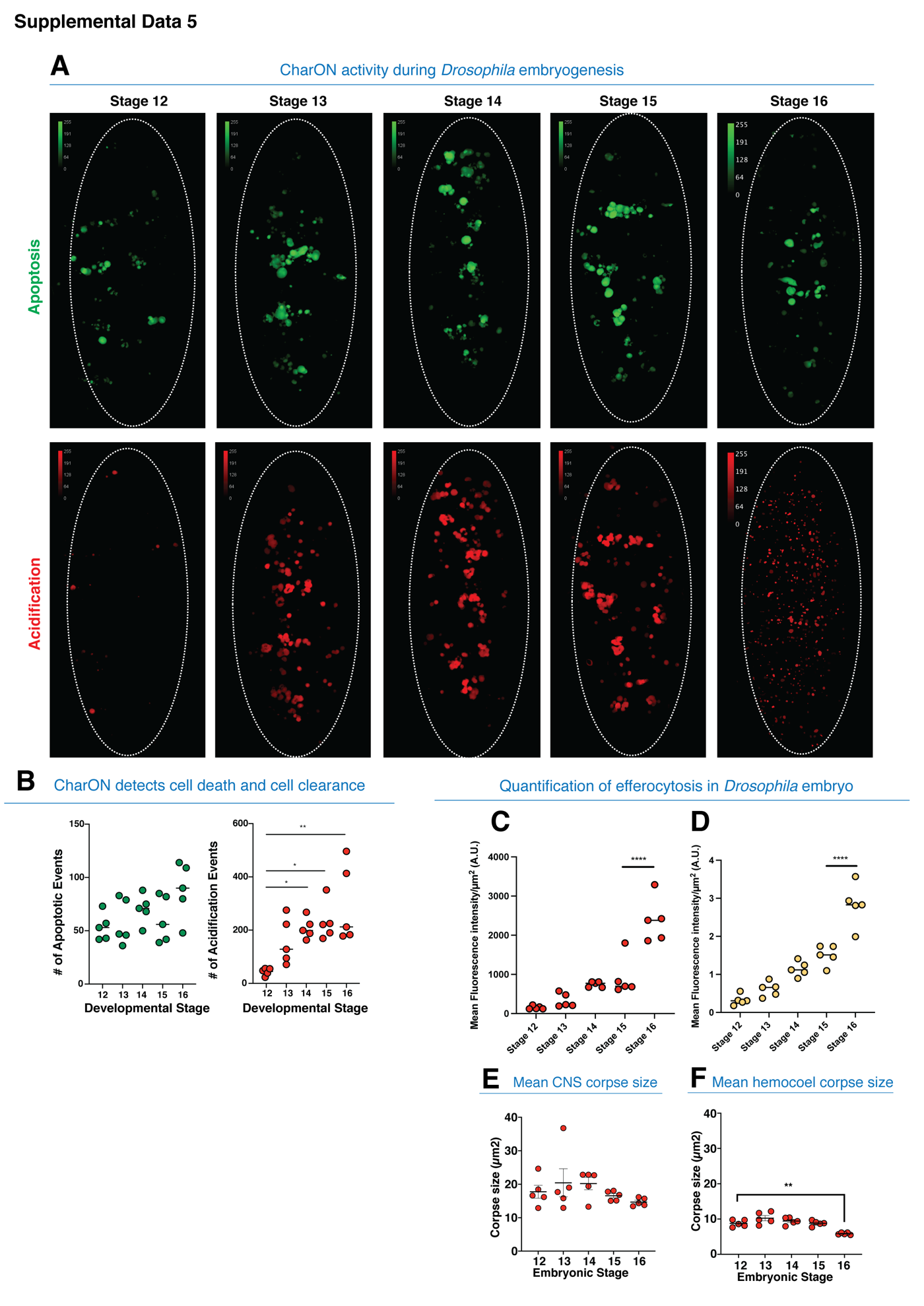


**Fig. S5: CharON is expressed in *Drosophila* and detects efferocytosis.** (**A**) Apoptosis (pH-CaspGFP) and Acidification (pHlorina) of CharON expressing *Drosophila* was evaluated within the embryonic CNS across stages 12 to 16. Events were thresholded and segmented using Ilastik. (**B**) (left) Quantification of Apoptosis (pH-CaspGFP); (right) Acidification (pHlorina) within CharON expressing *Drosophila* across stages 12 to 16 of embryogenesis. (One-Way ANOVA, ** = p<.0021, * = p<.0332). (**C**) Embryo-wide CharON expressing *Drosophila* RFP (pHlorina) signal during stages 12 through 16 of embryogenesis. N = 4 embryos. (**D**) Embryo-wide CharON expressing *Drosophila* CharON Ratio (pHlorina / pH-CaspGFP) signal during stages 12 to 16 of embryogenesis. N = 5 embryos. (**E**) Average size of corpses within the CNS region of the embryo from stages 12 to 16. (**F**) Average size of corpses within the hemocoel (blood cavity) region of the embryo from stages 12 to 16 (One-Way ANOVA, ** = p<.0021).

**Fig. S6**


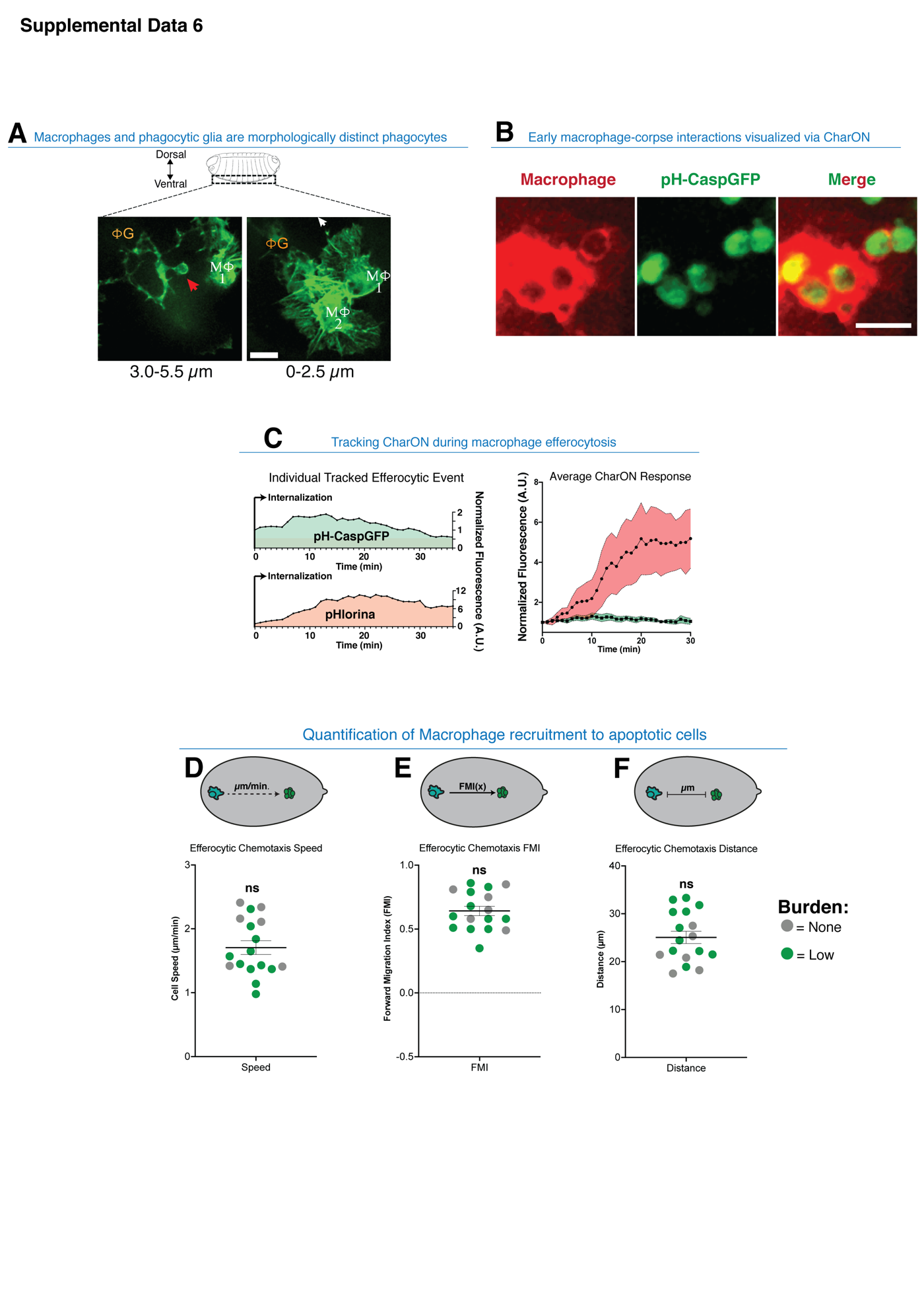


**Fig. S6: CharON reveals phagocyte dynamics during efferocytosis in vivo.** (**A**) Macrophages and phagocytic glia are morphologically distinct phagocytes. Phagocytic glia (ΦG) are non-motile, spindly phagocytes embedded in the CNS. The ventral-most (boxed region on embryo outline) phagocytic glia are in extremely close proximity to the highly motile macrophages (MΦ), both of which act together to clear the CNS of developmental apoptosis. Arrow highlights phagocytic filopod extended by glia during efferocytosis. Both images z-projections of indicated depths into embryo. Scale bars = 10 µm. (**B**) Early macrophage-corpse interactions can be optimally visualized through use of RFP-labelling of macrophages. pH-CaspGFP (green) of CharON labelled apoptotic corpses can be distinguished from RFP-labelled macrophages (red) during corpse engagement and uptake. Scale bars = 10 µm. (**C**) (left) Tracking of CharON fluorescence (pH-CaspGFP and pHlorina) during macrophage mediated efferocytosis. Upon internalization (T = 0 min), an event was tracked over 1 minute intervals. Fluorescence was normalised to T = 0 min. (Right) Fold change in mean normalised CharON fluorescence response during macrophage-mediated efferocytosis (=7 tracked corpses). (**D-F**) (Top) diagrams highlighting measured parameters. (Bottom) Quantification of *in vivo* efferocytic chemotaxis (stages 12-13). Grey data points indicate macrophages with no pre-exisiting corpse burden, green data points indicate macrophages with low (1-3 corpses) burden. Cell tracking during efferocytosis yielded (**D**) macrophage speed towards corpse (µm/min), (**E**) chemotactic accuracy (Forward Migration Index (FMI), wherein 0 = random migration and >0 = chemotaxis) towards corpses and (**F**) initial distance (µm) between macrophage and corpse (cell centroid-centroid). In all cases, no significant differences (ns) were detected between macrophages with no or low corpse burden (Unpaired t-test, ns = p>0.05, 17 cells from 6 embryos). Bars = mean ±S.E.M.

**Fig. S7**


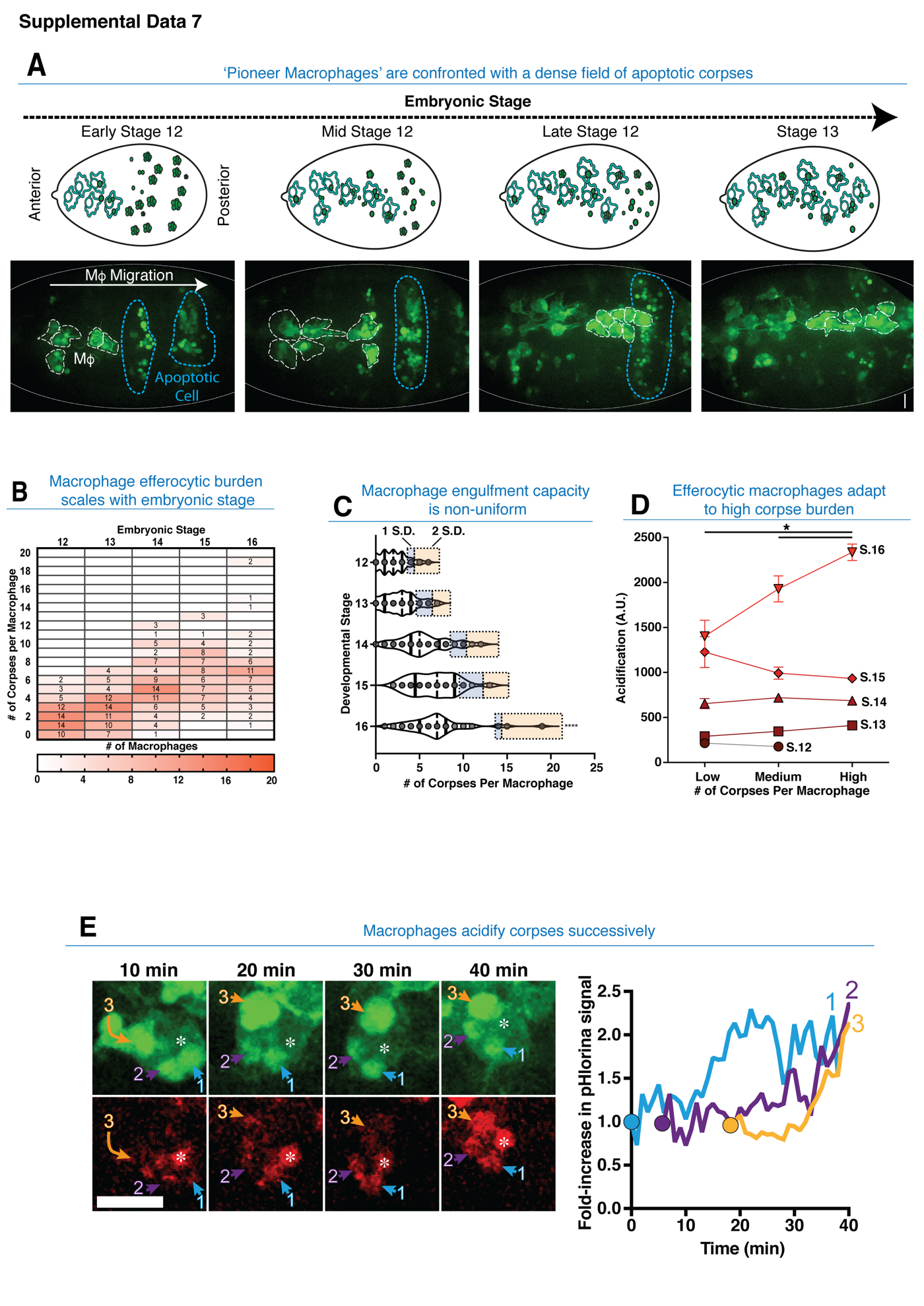


**Fig. S7: CharON reveals efferocytic heterogeneity in vivo.** (**A**) Diagrams and representative images highlighting how the first (‘pioneer’) GFP-labelled macrophages (MΦ, white dashed outlines) emerging on the ventral side of embryo (early-stage 12) are confronted by a high concentration of CharON-labelled apoptotic corpses (pH‑CaspGFP, blue dashed outlines). Embryo outlined in white, embryonic stages 12-13. Scale bar = 10 µm. (**B**) Absolute number of corpses /macrophages increases with increasing developmental time (embryonic stages 12-16, 5 embryos/stage). (**C**) The range in corpse number/macrophage is non-uniform (****) during late stage embryogenesis (stage 16). Macrophages with corpse burden 1 and 2 standard deviations from the mean are highlighted (blue and orange boxes respectively) for each embryonic stage (D’Agostino & Pearson Normality test, p<=0.0001, 5 embryos/stage). (**D**) Mean corpse acidification/macrophage increases uniformly during clearance (up to embryonic stage 15), regardless of corpse burden. However, by stage 16, macrophages with high corpse burdens (7+) have significantly (*) elevated mean corpse acidification/macrophage, implying adaptation to their increased burdens (One-Way ANOVA, * = p<0.0332, 5 embryos/stage). (**E**) The order in which corpses are engulfed match their acidification. (Left) Macrophage (GFP-moesin, green) efferocytosis of three successively engulfed CharON labelled corpses (pH-CaspGFP, green and pHlorina, red) was tracked over 40 minutes. Numbering indicates order of uptake (1-3, asterisks denote a previously engulfed corpse). RIGHT: Fold-increase in pHlorina signal (acidification) plotted for corpses 1-3, normalised to signal at point of uptake (coloured circles). Acidification of corpses occurs in the order they were engulfed. Scale bar = 10 µm.

**Fig. S8**


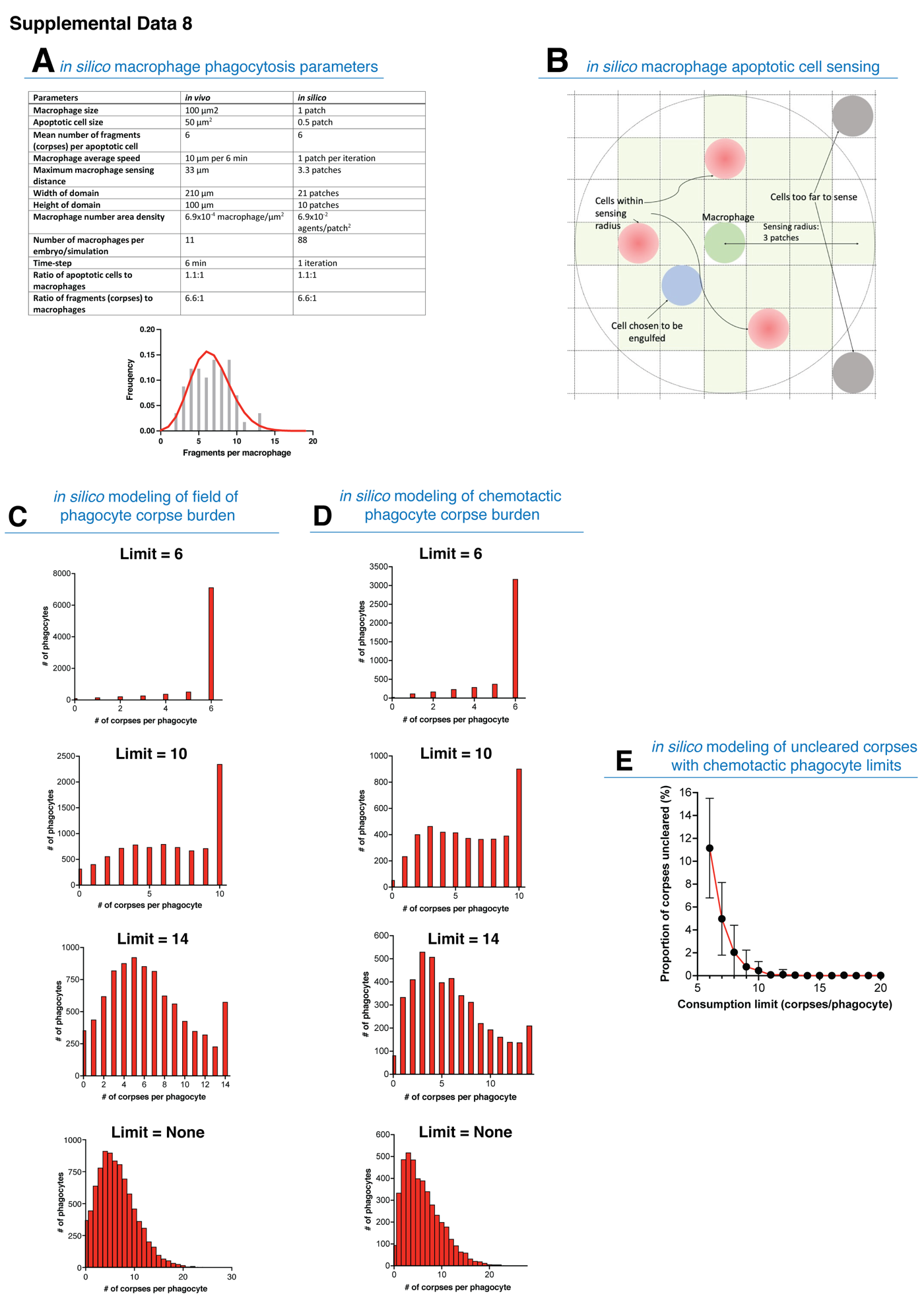


**Fig. S8: In silico modeling of macrophage efferocytosis.** (**A**) (Top) Table of *in silico* parameters used in mathematical modelling and the *in vivo* values they were derived from. (Bottom) The experimental burden per macrophage (at embryonic stage 15) is well described by a binomial distribution, implying that the fragments are randomly distributed amongst the macrophages. (**B**) Macrophage sensing and engulfment rule within the agent-base model. Macrophages sense cells which are available to be engulfed (red) within a sensing radius of 3 patches (green patches) and then choose the closest cell (blue) to engulf. Cells outside of this sensing radius (grey) are not considered. (**C**) Randomly distributed virtual phagocytes with varying capacity limits (6, 10, 14 or no limit, equal sharing of corpses =6/cell) were simulated moving with random-biased movement through a field of randomly distributed corpses. Varied and unequal burdens emerge when phagocytes are released from strict consumption limits, consistent with macrophage corpse burdens observed in vivo*.* (**D**) Unequal corpse burden arises in phagocytes chemotaxing through a field of corpses in silico. Virtual phagocytes with varying capacity limits (6, 10, 14 or no limit, equal sharing of corpses =6/cell) were simulated chemotaxing through a field of randomly distributed corpses. As with random distributed phagocytes, varied and unequal burdens emerge when chemotaxing phagocytes are released from strict consumption limits, consistent with macrophage corpse burdens observed in vivo*.* (**E**) In chemotaxis simulation, the efficiency of in silico corpse clearance by chemotaxing phagocytes is impaired if a strict consumption limit is enforced on the phagocytes (equal sharing of corpses =6/cell).

**Fig. S9**


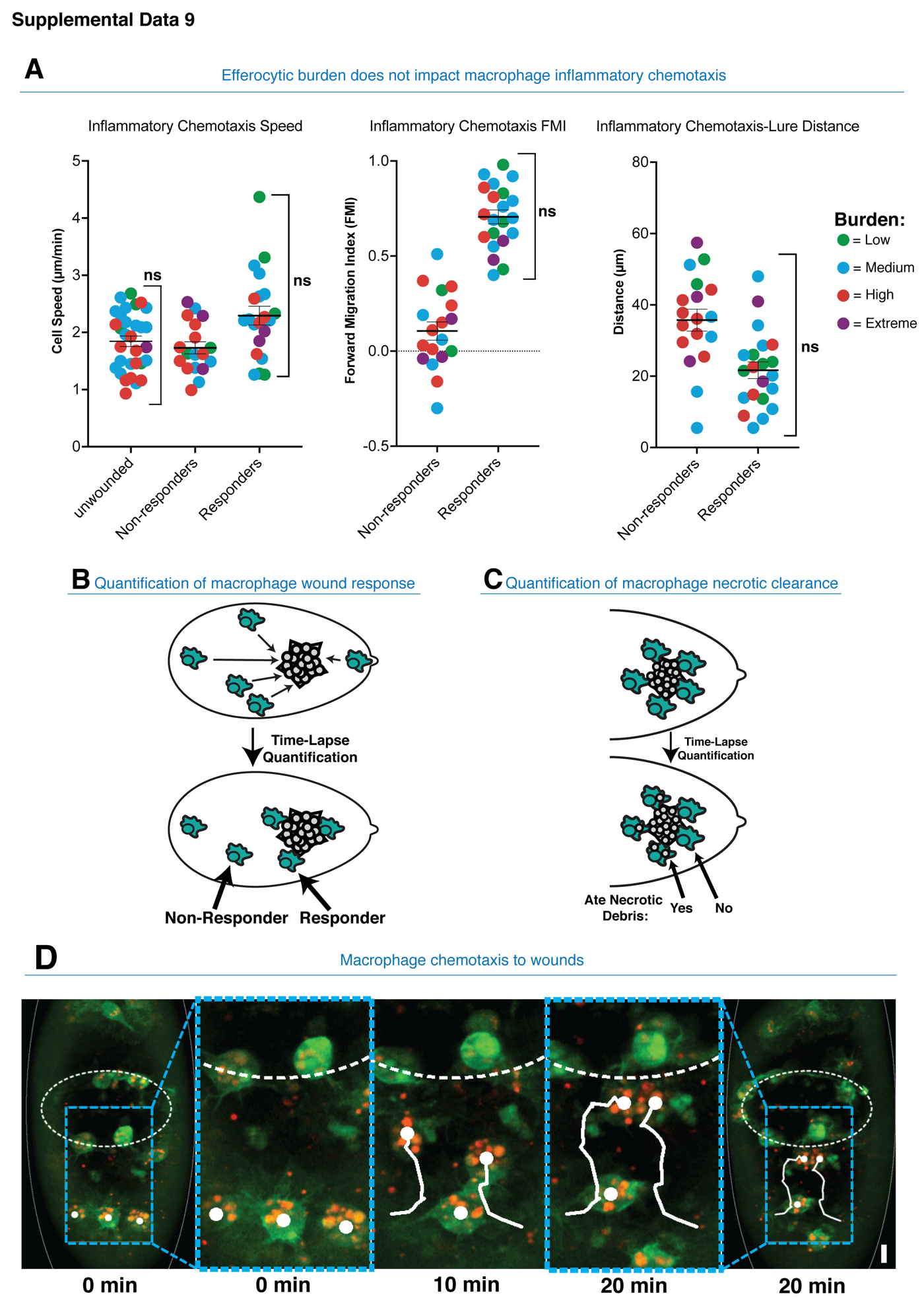


**Fig. S9: Evaluation of corpse burden on macrophage wound response.** (**A**) Macrophage inflammatory chemotaxis is not affected by corpse burden. After laser-induced wounding of stage 15 embryos (post-clearance), the motility of macrophages with varying corpse burdens was quantified through tracking (green data points = low corpse burden, blue data points = medium burden, red data points = high burden and purple data points = extreme burden, 5 embryos/genotype). (Left) Macrophages responding to the wound move with significantly increased speed compared to non-responders and basally migrating macrophages (in unwounded embryos). However, corpse burden has no significant affect (ns, One-way ANOVA) on cell speed. (Middle) Inflammatory macrophages chemotax towards wounds with high accuracy compared to non-responding cells (Forward Migration Index (FMI), wherein 0 = random migration and >0 = chemotaxis). However, corpse burden has no significant effect (ns, One-way ANOVA) on inflammatory chemotaxis. (Right) Macrophages responding to wounds are generally lured over a shorter distance than non-responding cells. Corpse burden does not significantly affect (ns, One-way ANOVA) the distance a macrophage will chemotax to the wound. (**B**) Schematic illustrating macrophage (green) recruitment to laser-induced wounds. (**C**) Schematic illustrating macrophage (green) recruitment to and clearance debris at laser-induced wounds. (**D**) After laser-induced wounding at stage 15 (post-clearance), macrophages (GFP-moesin, green) were recruited to the wound edge (dashed oval), and their apoptotic corpse burdens measured through CharON (green/red). Three macrophages with Medium-High corpse burdens that start equidistant from the wound edge (0 min), were tracked (white dots/lines) over 20 min. While one macrophage did not respond to the wound, high corpse burden does not appear to impede the chemotaxis of the other two macrophages. Scale bar =10 µm.

**Fig. S10**


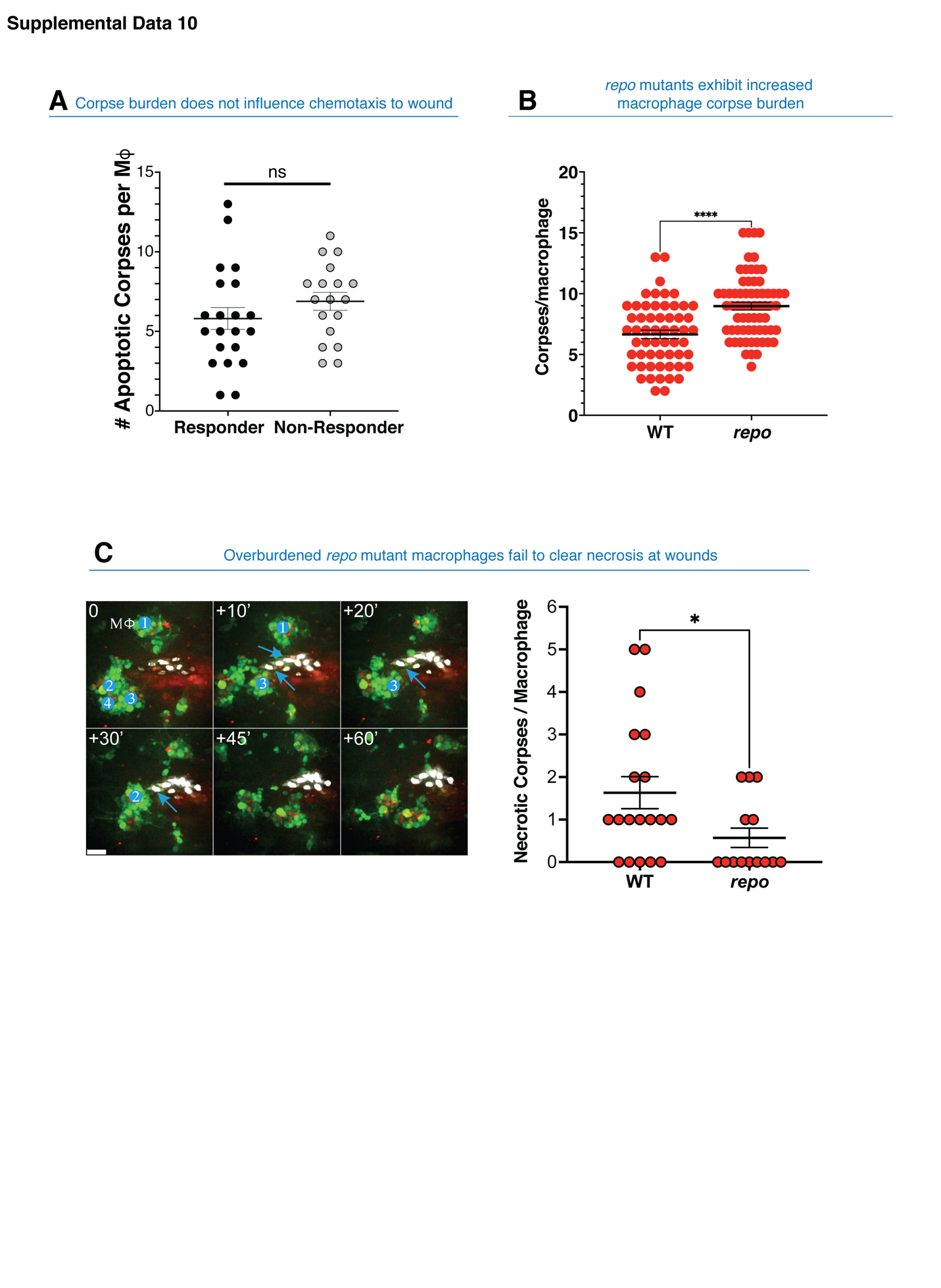


**Fig. S10: Increased corpse burden and impaired necrotic clearance in *repo* macrophages.** (**A**) Macrophage corpse burden does not impair their recruitment to wounds (Unpaired t-test, ns = p>0.05, 5 wounded embryos) Error bars = mean ±SEM. (**B**) Stage 15 *repo* mutants have significantly increased macrophage corpse burden compared to stage 15 wild-type (WT) controls (*** =p<0.0001, Unpaired t-test, 5 embryos/genotype). Error bars = mean ±S.E.M. (**C**) Overburdened *repo* mutant macrophages fail to clear necrosis at wounds. (Left) A necrotic stain (DRAQ7, white) was injected into *repo* mutant embryos with macrophage (MΦ and numbered blue circles) specific expression of GFP-moesin (green) and apoptotic corpses labelled with CharON (green/red). Following wounding through laser-ablation, *repo* mutant macrophages fail to engulf DRAQ7-labelled necrotic debris, even during direct contact (blue arrows). Note lack of any DRAQ7 uptake by macrophages even after 60 minutes. Time = minutes, scale bar =10 µm. (Right) Quantification of DRAQ7-labelled corpse uptake by recruited control (WT) and *repo* mutant macrophages. *repo* mutant macrophages engulf significantly (*) less necrotic debris compared to controls (Mann-Whitney Test, p=0.037, 3 embryos/genotype).

**Legends for Videos**

**Video S1: CharON visualises stages of efferocytosis *in vitro.*** CharON expressing Jurkat cells were rendered apoptotic and co-cultured with mouse macrophages. Efferocytosis was tracked through time-lapse imaging from target engagement to digestion. Time = hour: minutes, scale bar =20 µm.

**Video S2: CharON visualises multi-corpse engulfment *in vitro*.** CharON expressing Jurkat cells were rendered apoptotic and co-cultured with mouse macrophages. Multi-engulfment events were tracked via time-lapse imaging. In this case, a succession of corpses (arrows, first =green, second =blue, third =magenta) were engulfed by the one macrophage. Time = hour: minutes, scale =20 µm.

**Video S3: CharON visualises *in vivo* efferocytosis during *Drosophila* embryogenesis.** Ubiquitous expression of CharON in the *Drosophila* embryo revealed apoptosis (pH-CaspGFP, green) and efferocytosis (pHlorina, red) during Central Nervous System (CNS) development (embryonic stages 12-14), either within the CNS (top row) or superficial to the CNS and within the interstitial ‘blood-cavity’ (bottom row). Corpse motility in the latter is due to engulfment by macrophages. Examples of efferocytosis are highlighted with circles, and embryo outlines are shown in white. Time = minutes, scale bar =10 µm.

**Video S4: Macrophage migration and efferocytosis during *Drosophila* embryogenesis.** (Left) During embryogenesis, macrophages (GFP-moesin, green) migrate in an anterior to posterior direction along the ventral midline, encountering a dense field of apoptotic corpses (pH-CaspGFP, green). As revealed by CharON, macrophages rapidly engulf the apoptotic corpses, which are then acidified (pHlorina, red). (Right) A macrophage that obtains extreme corpse burden is tracked from source (white track). Engulfment events are circled. Embryonic stages 12-14, embryo outlined in white. Time = minutes, scale =10 µm.

**Video S5: Key stages of *in vivo* macrophage efferocytosis are revealed by CharON.** A CharON-labelled apoptotic cell (pH-CaspGFP, green, white arrow) attracts a macrophage (GFP-moesin, green), leading to engulfment. The internalised corpse and its acidification (pHlorina, red) can be tracked within the highly motile macrophage *in vivo*. Circled regions are shown in the right. Time = minutes, scale bar =10 µm.

**Video S6: Multiple macrophages often clear a single apoptotic cell.** A single CharON-labelled apoptotic cell (white arrow) fragments and is cleared by two different macrophages (GFP-moesin, cells distinguished with coloured dots). Time = minutes, scale bar =10 µm.

**Video S7: *In silico* efferocytosis impaired by enforced equal sharing of corpse burden.** Simulations of *in silico* corpse (red squares) clearance by *in silico* phagocytes (green dots), with either (left) no consumption limit or (right) a strict 6 corpse consumption limit (enforced equal sharing). A fixed number of virtual phagocytes and corpses are (top) randomly distributed and phagocytes move with a biased-random walk (random motility unless drawn towards a corpse until any consumption limit is reached, at which point they no longer engulf and move entirely randomly). Note that a consumption limit drastically increases the time taken for full clearance. (Bottom) Alternatively, a fixed number of virtual phagocytes chemotax through a fixed number of randomly distributed corpses. Phagocytes only deviate from chemotaxis to clear corpses (within a strict range), until any consumption limit is reached, at which point they are no longer attracted to corpses. Note that a consumption limit reduces the efficiency of clearance.

**Video S8: High corpse burden does not perturb the inflammatory recruitment of macrophages to wounds.** Shown are three macrophages (GFP-moesin, green, white dots) with high corpse burdens (CharON, green and red), which start equidistant to an epithelial wound (laser-ablation, dashed oval). Corpse burden does not appear to influence the inflammatory recruitment of macrophages to wounds since two out of three macrophages chemotax strongly toward the wound (white tracks). Embryo (stage 15) outlined in white. Time = minutes, scale bar = 10 µm.

**Video S9: Extreme corpse burden of *repo* mutant macrophages severely impairs uptake of necrotic debris and clearance of wounds.** Control (left) or *repo* mutant (right) embryos were injected with DRAQ7 (necrotic stain, white), wounded by laser-ablation and the resulting inflammation was visualized through 3-colour imaging. Developmental apoptotic corpses were labelled with CharON (pH-CaspGFP, green, and pHlorina, red) and macrophages were specifically labelled with GFP-moesin (green). While control macrophages rapidly engulf and clear necrotic debris, overburdened *repo* macrophages fail to engulf any necrotic debris. Time = minutes, scale bar =10 µm.

**Video S10: Macrophages with apoptotic corpse burdens efficiently engulf necrotic debris, promoting phagolysosomal fusion.** A macrophage (GFP-moesin, green) with a pre-existing burden of CharON-labelled apoptotic corpses (pH-CaspGFP, green, and pHlorina, red), engulfs necrotic debris (fluorescent-negative, filled arrow) at a wound (laser-ablation). This internalised necrotic corpse is rapidly acidified through fusion with a CharON-labelled apoptotic corpse (outlined arrow). Note that necrosis lacks caspase activation and therefore does not activate pH-CaspGFP. However, the necrotic corpse remains sensitive to acidification via pHlorina. Embryonic stage 15. Time = minutes, scale bar =10 µm.

**References**

1. S. Morioka, C. Maueröder, K. S. Ravichandran, Living on the Edge: Efferocytosis at the Interface of Homeostasis and Pathology. *Immunity*. **50**, 1149–1162 (2019).
2. J. M. Kinchen, K. S. Ravichandran, Phagosome maturation: going through the acid test. *Nature reviews. Molecular cell biology*. **9**, 781–95 (2008).
3. L. Fourgeaud, P. G. Través, Y. Tufail, H. Leal-Bailey, E. D. Lew, P. G. Burrola, et al., TAM receptors regulate multiple features of microglial physiology. *Nature.* **532**, 240–4 (2016).
4. N. A-Gonzalez, J. A. Quintana, S. García-Silva, M. Mazariegos, A. de la Aleja, J. A. Nicolás-Ávila, et al., *Journal of Experimental Medicine*, in press, doi:10.1084/jem.20161375.
5. P. Ayata, A. Badimon, H. J. Strasburger, M. K. Duff, S. E. Montgomery, Y.-H. E. Loh, et al., Epigenetic regulation of brain region-specific microglia clearance activity. *Nature neuroscience* (2018), doi:10.1038/s41593-018-0192-3.
6. L. Galluzzi, G. Melino, G. Kroemer, Molecular mechanisms of cell death: recommendations of the Nomenclature Committee on Cell Death 2018. *Cell Death & Differentiation*. **25**, 486–541 (2018).
7. Q. Zhang, A. Schepis, H. Huang, J. Yang, W. Ma, J. Torra, et al., Designing a Green Fluorogenic Protease Reporter by Flipping a Beta Strand of GFP for Imaging Apoptosis in Animals. *Journal of the American Chemical Society* (2019), doi:10.1021/jacs.8b13042.
8. T.-L. L. To, A. Schepis, R. Ruiz-González, Q. Zhang, D. Yu, Z. Dong, et al., Rational Design of a GFP-Based Fluorogenic Caspase Reporter for Imaging Apoptosis In Vivo. *Cell chemical biology*. **23**, 875–82 (2016).
9. J. Zhang, X. Wang, W. Cui, W. Wang, H. Zhang, et al., Visualization of caspase-3-like activity in cells using a genetically encoded fluorescent biosensor activated by protein cleavage. *Nature Communications*. **4**, 2157 (2013).
10. TM Roberts, F. Rudolf, A. Meyer, R. Pellaux, E. Whitehead, S. Panke, et al., Identification and Characterisation of a pH-stable GFP. *Scientific Reports*. **6**, 28166 (2016).
11. N. C. Shaner, M. Z. Lin, M. R. McKeown, P. A. Steinbach, K. L. Hazelwood, M. W. Davidson, et al., Improving the photostability of bright monomeric orange and red fluorescent proteins. *Nat Methods*. **5**, 545–551 (2008).
12. Z. Liu, O. Chen, B. J. Wall, M. Zheng, Y. Zhou, L. Wang, et al., Systematic comparison of 2A peptides for cloning multi-genes in a polycistronic vector. *Scientific Reports.* **7**, 2193 (2017).
13. J. M. Abrams, K. White, L. I. Fessler, H. Steller, Programmed cell death during Drosophila embryogenesis. *Dev Camb Engl*. **117**, 29–43 (1993).
14. A. Rogulja-Ortmann, K. Lüer, J. Seibert, C. Rickert, G. M. Technau, Programmed cell death in the embryonic central nervous system of Drosophila melanogaster. *Dev Camb Engl.* **134**, 105–16 (2007).
15. A. J. Davidson, W. Wood, Phagocyte Responses to Cell Death in Flies. *Csh Perspect Biol*. **12**, a036350 (2020).
16. W. Wood, C. Faria, A. Jacinto, Distinct mechanisms regulate hemocyte chemotaxis during development and wound healing in Drosophila melanogaster. *J Cell Biol*. **173**, 405–416 (2006).
17. E. Kurant, S. Axelrod, D. Leaman, U. Gaul, Six-Microns-Under Acts Upstream of Draper in the Glial Phagocytosis of Apoptotic Neurons. *Cell.* **133**, 498–509 (2008).
18. A. J. Davidson, W. Wood, Macrophages Use Distinct Actin Regulators to Switch Engulfment Strategies and Ensure Phagocytic Plasticity In Vivo. *Cell Reports*. **31**, 107692 (2020).
19. T. A. Markow, S. Beall, L. M. Matzkin, Egg size, embryonic development time and ovoviviparity in Drosophila species: Ovoviviparity in Drosophila species. *J Evolution Biol*. **22**, 430–434 (2008).
20. Y. Wang, M. Subramanian, A. Yurdagul, V. C. Barbosa-Lorenzi, B. Cai, J. de Juan-Sanz, et al., Mitochondrial Fission Promotes the Continued Clearance of Apoptotic Cells by Macrophages. *Cell.* **171**, 331-345.e22 (2017).
21. A. Yurdagul, M. Subramanian, X. Wang, S. B. Crown, O. R. Ilkayeva, L. Darville, et al., Macrophage Metabolism of Apoptotic Cell-Derived Arginine Promotes Continual Efferocytosis and Resolution of Injury. *Cell Metabolism* (2020), doi:10.1016/j.cmet.2020.01.001.
22. N. A-Gonzalez, S. J. Bensinger, C. Hong, S. Beceiro, M. N. Bradley, N. Zelcer, et al., Apoptotic Cells Promote Their Own Clearance and Immune Tolerance through Activation of the Nuclear Receptor LXR. *Immunity*. 245–258 (2009).
23. W. C. Xiong, H. Okano, N. H. Patel, J. A. Blendy, C. Montell, repo encodes a glial-specific homeo domain protein required in the Drosophila nervous system. *Gene Dev*. **8**, 981–994 (1994).
24. G. Trébuchet, P. B. Cattenoz, J. Zsámboki, D. Mazaud, D. E. Siekhaus, M. Fanto, et al., The Repo Homeodomain Transcription Factor Suppresses Hematopoiesis in Drosophila and Preserves the Glial Fate. *J Neurosci*. **39**, 238–255 (2019).
25. E. L. Armitage, H. G. Roddie, I. R. Evans, Overexposure to apoptosis via disrupted glial specification perturbs Drosophila macrophage function and reveals roles of the CNS during injury. *Cell Death Dis*. **11**, 627 (2020).
26. T.-W. Chen, T. J. Wardill, Y. Sun, S. R. Pulver, S. L. Renninger, A. Baohan, et al., Ultrasensitive fluorescent proteins for imaging neuronal activity. *Nature.* **499**, 295–300 (2013).
27. A. S. Abdelfattah, T. Kawashima, A. Singh, O. Novak, H. Liu, Y. Shuai, et al., Bright and photostable chemigenetic indicators for extended in vivo voltage imaging. *Science (New York, N.Y.)*. **365**, 699–704 (2019).
28. H. G. Roddie, E. L. Armitage, J. A. Coates, S. A. Johnston, I. R. Evans, Simu-dependent clearance of dying cells regulates macrophage function and inflammation resolution. *PLOS Biology*. **17**, e2006741 (2019).
29. D. Park, C. Z. Han, M. R. Elliott, J. M. Kinchen, P. C. Trampont, S. Das, et al., Continued clearance of apoptotic cells critically depends on the phagocyte Ucp2 protein. *Nature.* **477**, 220–224 (2011).
30. A. C. Doran, A. Yurdagul, I. Tabas, Efferocytosis in health and disease. *Nature Reviews Immunology*, 1–14 (2019).
31. I. R. Evans, J. Zanet, W. Wood, B. M. Stramer, Live Imaging Of Drosophila melanogaster Embryonic Hemocyte Migrations. *J Vis Exp Jove*, 1696 (2010).
32. W. R. Carmody, Easily prepared wide range buffer series. *J Chem Educ*. **38**, 559
33. W. Wood, A. Jacinto, R. Grose, S. Woolner, J. Gale, C. Wilson, et al., Wound healing recapitulates morphogenesis in Drosophila embryos. *Nat Cell Biol*. **4**, 907–912 (2002).
34. T. Le, Z. Liang, H. Patel, M. H. Yu, G. Sivasubramaniam, M. Slovitt, G. Tanentzapf, et al., A New Family of Drosophila Balancer Chromosomes With a w− dfd-GMR Yellow Fluorescent Protein Marker. *Genetics*. **174**, 2255–2257 (2006).
35. M. Tantama, Y. Hung, G. Yellen, Imaging Intracellular pH in Live Cells with a Genetically Encoded Red Fluorescent Protein Sensor. *Journal of the American Chemical Society.* 133, 10034–10037 (2011).
36. V. Harteinstein, Atlas of Drosophila Development. *Cold Spring Harbor Laboratory Press* (1993).
37. A. Wodarz, U. Hinz, M. Engelbert, E. Knust, Expression of crumbs confers apical character on plasma membrane domains of ectodermal epithelia of drosophila. Cell. **82**, 67-76 (1995).
38. A. Gyoergy, M. Roblek, A. Ratheesh, K. Valoskova, V. Belyaeva, S. Wachner, et al., Tools Allowing Independent Visualization and Genetic Manipulation of *Drosophila* *melanogaster* Macrophages and Surrounding Tissues. *G3 (Bethesda)*. 8, 845-857 (2018).
